# Chloropicophyceae, a new class of picophytoplanktonic prasinophytes

**DOI:** 10.1038/s41598-017-12412-5

**Published:** 2017-10-25

**Authors:** Adriana Lopes dos Santos, Thibaut Pollina, Priscillia Gourvil, Erwan Corre, Dominique Marie, José Luis Garrido, Francisco Rodríguez, Mary-Hélène Noël, Daniel Vaulot, Wenche Eikrem

**Affiliations:** 1 Sorbonne Universités, UPMC Université Paris 06, CNRS, UMR7144, Station Biologique de Roscoff, Roscoff, France; 2 Norwegian Institute for Water Research, Gaustadalléen 21, 0349, Oslo, Norway; 3 Instituto de Investigaciones Marinas (CSIC). Av. Eduardo Cabello, 6. 36208, Vigo, Spain; 4 Instituto Español de Oceanografia (IEO), Centro Oceanográfico de Vigo, Subida a Radio Faro, 36390 Vigo, Spain; 5 Centro de Genómica y Bioinformática, Facultad de Ciencias, Universidad Mayor. Camino La Pirámide 5750, Huechuraba, Santiago Chile; 6 National Institute for Environmental Studies, Tsukuba, Japan; 7 Natural History Museum, University of Oslo, PO Box 1069, Blindern, 0316 Oslo Norway; 8 Department of Biosciences, University of Oslo, PO Box 1066, Blindern, 0316 Oslo Norway

**Keywords:** Biodiversity, Marine biology

## Abstract

Prasinophytes are a paraphyletic group of nine lineages of green microalgae that are currently classified either at the class or order level or as clades without formal taxonomic description. Prasinophyte clade VII comprises picoplanktonic algae that are important components of marine phytoplankton communities, particularly in moderately oligotrophic waters. Despite first being cultured in the 1960s, this clade has yet to be formally described. Previous phylogenetic analyses using the 18S rRNA gene divided prasinophyte clade VII into three lineages, termed A, B and C, the latter formed by a single species, *Picocystis salinarum*, that to date has only been found in saline lakes. Strains from lineages A and B cannot be distinguished by light microscopy and have very similar photosynthetic pigment profiles corresponding to the prasino-2A pigment group. We obtained phenotypic and genetic data on a large set of prasinophyte clade VII culture strains that allowed us to clarify the taxonomy of this important marine group. We describe two novel classes, the Picocystophyceae and the Chloropicophyceae, the latter containing two novel genera, *Chloropicon* and *Chloroparvula*, and eight new species of marine picoplanktonic green algae.

## Introduction

Prasinophytes are a paraphyletic group of nine lineages of green microalgae that are currently classified either at the class or order level or family or as clades without formal taxonomic description^1^. The taxonomy of prasinophytes has proved particularly challenging in part due to the small size and simple morphology of many of its members^2,3^. A good example of this is prasinophyte clade VII^4^ that are coccoid cells ranging in size from 2 to 3 µm with few specific morphological features.

Phylogenetic analysis of the 18S rRNA gene divided prasinophyte clade VII into three lineages, termed A, B and C^4^, the latter formed only by *Picocystis salinarum*, a picoplanktonic species described from saline lakes^5–7^. Strains from lineages A and B cannot be distinguished by light microscopy and have very similar photosynthetic pigment profiles corresponding to the prasino-2A pigment group^8^. *P*. *salinarum* cells tend towards an easily observed tri-lobed shape under conditions of nutrient depletion and possess monadoxanthin and diatoxanthin as major carotenoids^5^. In contrast to results from phylogenetic analyses using the 18S rRNA gene, analyses using partial plastid 16S rRNA gene sequences^9^, complete nuclear and plastid encoded rRNA operons^10,11^ and chloroplast genomes^12^ suggest that *P*. *salinarum* forms a lineage that is separate from prasinophyte clade VII A and B. In the absence of morphological differentiation, molecular data obtained from culture strains and environmental samples have allowed the delimitation of at least 10 different phylogenetic clades, termed A1 to A7 and B1 to B3^9^, within prasinophyte clade VII.

From an ecological point of view, prasinophyte clade VII appears to be a major group of picoplanktonic green algae in marine waters^9,13–17^. In moderately oligotrophic areas it is often the main Chlorophyta group, replacing Mamiellophyceae which tends to dominate in coastal waters^9,14^. Clades B1 and A4 typically dominate in oceanic waters and different sub-clades seem to occupy distinct niches, although the precise habitat of each clade is still unclear^9^.

Prasinophyte clade VII remains without formal taxonomic description despite the fact that members of this clade have been maintained in culture since 1965^18^. In recent years the principle of combining morphological and molecular data to delineate species has increasingly been adopted in microalgal taxonomy. Intra- and inter-specific genetic variation in molecular markers are used to describe individuals and determine DNA-based species^19^. In addition to sequence divergence methods, analysis of the secondary structure of the Internal Transcribed Spacer 2 (ITS2) has been used for delimiting biological species. The ITS2 is part of the eukaryotic nuclear ribosomal operon, located between the 5.8S and 28S rRNA genes (ITS1 is located between the 18S and 5.8S rRNA genes). The primary sequence and length of ITS2 vary extensively among different taxa, however its secondary structure, when transcribed into RNA, retains features that are important for its biological function and thought to be universal among eukaryotes^20–23^. To generate new rRNA molecules, the entire operon is transcribed as a single rRNA precursor and the new 18S and 28S rRNA molecules are obtained after a complex excision process of both ITS regions primarily guided by their transcripts secondary structure^24,25^. The use of secondary structure of ITS2 in microalgal taxonomy increased after Coleman *et al*.^21^ and Muller *et al*.^26^ suggested a link between the presence of compensatory base changes (CBCs) and species boundaries. The ITS2 secondary structure includes four helices. A double-sided base change of a nucleotide pair in a given helix retaining the secondary structure is considered a CBC, while a single-side change is called hemi-CBC (hCBC).

In this paper, we analyze a wide set of phenotypic and genetic characters of members of prasinophyte clade VII, including ultrastructure, cell size, DNA content, pigment profiles, multigene phylogeny and ITS2 secondary structure. The data obtained lead us to describe two novel classes, the Picocystophyceae and the Chloropicophyceae, which contain two novel genera, *Chloropicon* and *Chloroparvula*, and eight species that are new to science.

## Material and Methods

### Cultured strains

The prasinophyte clade VII strains used in this study are listed in Table 1. These strains were selected from the Roscoff Culture Collection (RCC, http://www.roscoff-culture-collection.org) and Microbial Culture Collection at NIES (National Institute for Environmental Studies, http://mcc.nies.go.jp). Strains were grown at 22 °C in L1 seawater medium^27^ under an average light intensity of 100 µmoles photons.m^−2^.s^−1^ and a 12:12 h LD (Light:Dark) regime. NIES strains were grown in ESM seawater medium^28^.

**Table 1:** Strains of prasinophytes clade VII used in this study.

Strain ID	Species	Strain name	Other name	Clade	Ocean origin	Latitude	Depth of isolation (m)	Isolation date	18S rRNA	16S rRNA	ITS
RCC712		IndianOcean_36-1		A1	Indian Ocean	−22.08	120	29-May-2003	KU843579	LN735451	MF077495
RCC713		IndianOcean_37-2		A1	Indian Ocean	−22.08	120	29-May-2003	KU843580	LN735452	MF077496
RCC719		IndianOcean_45-8		A1	Indian Ocean	−12.22	76	7-Jun-2003	KU843582	LN735454	MF077497
RCC997		Biosope_46 B5S	NIES-2675	A1	Pacific Ocean	−9.07	100	2-Nov-2004	KT860935	LN735515	MF077503
**RCC998**	***Chloropicon mariensis***	**Biosope_46 C3S**	**NIES-2676**	**A1**	**Pacific Ocean**	**−9**.**07**	**100**	**2-Nov-2004**	**KF422632**	**LN735516**	**MF077504**
RCC138		CCMP1606		A2	Pacific Ocean	22.75	NA	1-Jan-1992	KT860872	LN735241	MF077488
**RCC15**	***Chloropicon primus***	**CCMP1205**		**A2**	**Atlantic Ocean**	**NA**	**NA**	**1-Jul-1965**	**U40921**	**AY702121**, **FN563080**	**HE610139**
RCC717		IndianOcean_43-5		A2	Indian Ocean	−14.48	0	11-Jun-2003	KU843581	LN735453	MF077479
NIES-3671		CREST MH 514		A3	Pacific Ocean	38.01	0	3-Oct-2012	KU843576	KU843562	MF077477
RCC1019		Biosope_45 A2 478		A3	Pacific Ocean	−9.07	100	2-Nov-2004	KU843588	LN735204	MF077506
RCC1032		Biosope_46 B7		A3	Pacific Ocean	−9.07	100	2-Nov-2004	KU843590	LN735207	MF077507
RCC1043		Biosope_47 B1		A3	Pacific Ocean	−9.07	30	2-Nov-2004	KU843591	LN735210	MF077508
**RCC287**	***Chloropicon sieburthii***	**NOUM15**	**NOUM97015**	**A3**	**Pacific Ocean**	**0**.**00**	**120**	**10-Feb-1998**	**AY425302**	**AY702147**	**MF077508**
RCC297		Açores 3		A3	Atlantic Ocean	38.83	0	19-Mar-1998	KT860659	LN735413	MF077491
RCC857		Biosope_40 A2		A3	Pacific Ocean	−8.33	10	29-Oct-2004	KU843585	LN735471	MF077500
NIES-2755		JST MH 317	RCC2335	A4	Pacific Ocean	35.22	0	4-May-2009	KF422627	LN735348	MF077511
NIES-3667		CREST MH 504	RCC3373	A4	Pacific Ocean	37.98	0	1-Sep-2012	KU843594	KU843566	MF077514
NIES-3668		CREST MH 537		A4	Pacific Ocean	38.03	0	1-Sep-2012	KU843573	KU843559	MF077513
NIES-3670		CREST MH 533		A4	Pacific Ocean	42.16	0	3-Oct-2012	KU843575	KU843561	MF077487
RCC1124		PAP_AD		A4	Atlantic Ocean	48.83	10	6-Jul-2006	KU843592	LN735219	MF077509
**RCC1871**	***Chloropicon roscoffensis***	**RA090205-09**		**A4**	**English Channel**	**48**.**75**	**0**	**5-Feb-2009**	**KF899840**	**LN735295**	**MF077510**
RCC4429		AMT 2013 - P180-A5		A4	Atlantic Ocean	44.11	2	10-Oct-2013	KU843597	KU843571	MF077517
RCC4430		AMT 2013 - P181-A1		A4	Atlantic Ocean	44.11	2	10-Oct-2013	KU843598	KU843572	MF077518
RCC722		IndianOcean_47-2		A4	Indian Ocean	−12.22	6	7-Jun-2003	KU843583	KU843566	MF077498
RCC726		IndianOcean_49-8		A4	Indian Ocean	−14.48	0	11-Jun-2003	KU843584	LN735455	MF077499
RCC917		Biosope_182_FL1-3		A4	Pacific Ocean	−33.35	5	4-Dec-2004	FJ997211	LN735488	MF077501
RCC1021		Biosope_46 B6		A5	Pacific Ocean	−9.07	100	2-Nov-2004	KU843589	LN735205	strain losted
RCC19		OLI 26 FG-B		A5	Pacific Ocean	−7.00	60	10-Nov-1994	KT860855	KU843563	MF077478
RCC227		OLI 26 FB-A		A5	Pacific Ocean	−7.00	60	10-Nov-1994	KT860875	KU843564	MF077489
RCC3375		CCMP2175		A5	Pacific Ocean	22.75	NA	21-Sep-1992	KF899843	KU843569	MF077516
RCC700		IndianOcean_6-3		A5	Indian Ocean	−14.48	70	11-Jun-2003	KU843578	LN735441	MF077493
RCC701		IndianOcean_8-1		A5	Indian Ocean	−14.48	70	11-Jun-2003	KF899839	KU843565	MF077494
**RCC856**	***Chloropicon laureae***	**Biosope_42 A2**		**A5**	**Pacific Ocean**	**−8**.**33**	**10**	**29-Oct-2004**	**KF422631**	**LN735470**	**MF077480**
RCC887		IndianOcean_8-2-C4	RCC702-C4	A5	Indian Ocean	−14.48	70	11-Jun-2003	MF077474	MF077471	MF077481
RCC4434		AMT 2013 - P182-H9		A6	Atlantic Ocean	17.06	2	18-Oct-2013	KU843599	MF077472	MF077519
**RCC3374**	***Chloropicon maureeniae***	**CCMP2152**		**A7**	**Pacific Ocean**	**22**.**75**	**NA**	**24-Nov-1994**	**KU843595**	**KU843568**	**MF077515**
RCC3368		CCMP2111		A	Indian Ocean	−5.47	NA	9-Oct-1994	MF077475	LN735423	MF077513
RCC3376		CCMP2113		A	Pacific Ocean	8.93	85	1-Sep-1991	KU843596	KU843570	MF077483
RCC996		Biosope_46 B4S		A	Pacific Ocean	−9.07	100	2-Nov-2004	KU843586	LN735514	MF077502
**NIES-3669**	***Chloroparvula pacifica***	**CREST MH 509**	**RCC4656**	**B1**	**Pacific Ocean**	**42**.**16**	**0**	**3-Oct-2012**	**KU843574**	**KU843560**	**MF077502**
NIES-2756		JST MH 335	RCC2337	B2	Pacific Ocean	33.77	0	29-Jun-2009	KU843593	LN735349	MF077512
RCC696		IndianOcean_1-1		B2	Indian Ocean	−22.08	120	29-May-2003	KU843577	LN735439	MF077492
RCC999		Biosope_46 C4S		B2	Pacific Ocean	−9.07	100	2-Nov-2004	KU843587	LN735517	MF077505
RCC4572		AMT 2014 FLG46-1		B3	Atlantic Ocean	−29.15	**2**	22-Oct-2014	MF077476	MF077473	
**NIES-2758**	***Chloroparvula japonica***	**JST MH 340**	**RCC2339**	**B**	**Pacific Ocean**	**33**.**77**	**0**	**29-Jun-2009**	**KF422628**	**LN735350**	**MF077482**
RCC3402	*Picocystis salinarum*	CCMP1897		C	San Francisco salt pond	37.78	NA	5-Dec-1999	FR865649	AB491631	HE610138, MF077484

RCC: Roscoff Culture Collection (www.roscoff-culture-collection.org). NIES: National Institute for Environmental Studies, Microbial Culture Collection (http://mcc.nies.go.jp). NA: data not available. Strains in bold correspond to authentic strains used to describe the new cultures.

### Pigments Analysis

Approximately 50 ml of cultures listed in Table 1 (except NIES-3669 which required 200 ml) were collected in late exponential or early stationary phase by filtration onto glass fiber GF/F filters (Whatman, Maidstone, UK) without applying vacuum. Prior to sample collection, cell concentration was determined by flow cytometry using a Becton Dickinson Accuri C6. Total time for filtration did not exceed 10 minutes and total volume filtered was recorded. Filters were removed as soon as they became clogged, protected from light at all processing stages and immediately frozen in liquid nitrogen and stored at −80 °C. Frozen filters were extracted with 3 mL of 90% acetone in screw cap glass tubes with polytetra-fluoroethylene (PTFE) lined caps, placed in an ice-water bath. After 15 minutes, filters were homogenized using a clean stainless steel spatula for filter grinding. Tubes were placed in an ultrasonic bath with water and ice for 5 minutes. The slurries were then centrifuged for 5 minutes at 3940 g and supernatants filtered through 13 mm diameter polypropylene syringe filters (MS PTFE, 0.22 µm pore size) to remove cell and filter debris. Before injection, 0.4 ml of Milli-Q water was added to 1 ml of each sample extract to avoid peak distortion. Pigments extracted from clade VII strains were analyzed using the method of Zapata *et al*.^29^ as modified by Garrido *et al*.^30^ to improve the separation of loroxanthin and neoxanthin (Table 2).

**Table 2:** Concentration of Chl *a* per cell and ratios (mol.mol^−1^) of pigment to Chl *a* concentration of 22 strains of prasinophytes clade VII grown under an average of 100 µmoles photons.m^−2^.s^−1^ New data are presented along with data from a previous study^8^.

Strain	Species	sub - clade	Light	fg Chl *a*/cell	Chl *b*	Chlide *a*	Chlide *b*	Loro	Neo	Viola	Asta	Anth	Zea	Lut	ββ - car	βε - car	Allo	Diato	Monado
RCC719*	***C***. ***mariensis***	A1	140	14.89	0.683	0	0	0.035	0.086	0.617	0.264	0	0.478	0.193	0.149	0.107	0	0	0
RCC998*		A1	140	26.07	0.783	0	0	0.014	0.117	0.877	0.157	0.058	0.168	0.285	0.131	0.095	0	0	0
RCC15 *	***C***. ***primus***	A2	140	20.34	0.901	0.066	0.018	0.058	0.003	0.327	0.165	0.043	0.103	0.376	0.093	0.154	0	0	0
RCC287*	***C***. ***sieburthii***	A3	140	4.99	0.986	0.106	0	0.024	0.167	0.572	0.210	0.024	0.043	0.363	0.079	0.051	0	0	0
RCC857*		A3	140	4.10	1.000	0	0	0.026	0.122	0.534	0.187	0.024	0.094	0.291	0.064	0.056	0	0	0
RCC1124*	***C***. ***roscoffensis***	A4	140	8.61	0.960	0.099	0	0	0.116	0.525	0.148	0.020	0.086	0.399	0.082	0.095	0	0	0
RCC1871*		A4	100	3.30	1.196	0	0	0	0.119	0.460	0.064	0.015	0.091	0.444	0.025	0.078	0	0	0
RCC917		A4	100	18.49	1.081	0.025	0.006	0	0.132	0.603	0.110	0.006	0.046	0.388	0.046	0.078	0	0	0
RCC726		A4	100	7.73	0.956	0.020	0	0	0.118	0.537	0.151	0.010	0.120	0.369	0.067	0.088	0	0	0
NIES-2755		A4	100	10.01	1.125	0.011	0	0	0.133	0.608	0.110	0.005	0.036	0.359	0.044	0.075	0	0	0
RCC4429		A4	100	10.62	1.044	0.027	0	0	0.118	0.697	0.115	0.010	0.038	0.373	0.052	0.094	0	0	0
NIES-3667		A4	100	30.17	1.108	0.024	0.013	0	0.129	0.570	0.152	0.006	0.044	0.354	0.067	0.070	0	0	0
RCC856*	***C***. ***laureae***	A5	140	23.37	0.860	0	0	0.113	0.079	0.291	0.570	0.042	0.310	0.461	0.136	0.075	0	0	0
RCC3374*	***C***. ***maureeniae***	A7	140	4.14	0.726	0.229	0	0.012	0.082	0.272	0.778	0.041	0.118	0.332	0.146	0.059	0	0	0
RCC996*		A	140	51.57	0.931	0.074	0	0.043	0.095	0.119	0.191	0.039	0.245	0.36	0.074	0.09	0	0	0
RCC3376 *		A	140	4.08	0.813	0.105	0	0.008	0.112	0.572	0.324	0.054	0.129	0.048	0.194	0.085	0	0	0
**NIES-3669**	***C***. ***pacifica***	B1	100	6.34	0.907	0	0	0	0.115	0.252	0	0.153	0.298	0.766	0	0.107	0	0	0
RCC696		B2	100	26.30	1.030	0.061	0.042	0	0.125	0.569	0.095	0.009	0.050	0.437	0.081	0.058	0	0	0
NIES-2756*		B2	140	4.37	0.882	0.394	0.236	0.031	0.137	0.504	0.302	0.051	0.154	0.706	0.240	0.061	0	0	0
RCC4572		B3	100	27.19	1.032	0.130	0.100	0	0.136	0.534	0.108	0.006	0.019	0.454	0.088	0.055	0	0	0
NIES-2758*	***C***. ***japonica***	B	140	14.58	0.624	0	0	0.026	0.078	0.613	0.045	0.024	0.074	0.302	0.330	0.028	0	0	0
RCC3402*	***P***. ***salinarum***	C	100	60.40	0.283	0	0	0	0.039	0.035	0	0.003	0.018	0.071	0.129	0.015	0.047	0.107	0.119

*Reference: Lopes dos Santos *et al*.^8^

Carotenoids ratios to Chl *a* content.

### Light microscopy

Two milliliters of cultures in exponential or early stationary phase were harvested by centrifugation (2000 g, 5 minutes) and observed with light microscopy under an Olympus BX 51 microscope equipped with differential interference contrast (DIC), phase contrast and blue fluorescence filters. Microphotographs were obtained with a SPOT RT-slider digital camera (Diagnostics Instruments, Sterling Heights, MI). For cell size, about 100 randomly chosen cells were measured using the Fiji open - source platform^31^.

### Transmission electron microscopy

For thin sections, cells were fixed in 2% glutaraldehyde (final concentration) in growth medium for 1 h at room temperature and centrifuged (4000 rpm, 30 min) to form a pellet that was rinsed three times in growth medium (5 min each) and then three times in 0.1 M sodium cacodylate (5 min each). The cells were post-fixed in a mixture of 1% osmium tetroxide and 1% potassium ferricyanide in 0.1 M sodium cacodylate (final concentrations) for 2 hours at 4 °C and subsequently rinsed three times (10 min each) in 0.1 M cacodylate and twice in MilliQ water (5 min each). The cells were stained for 1 h in 1% aqueous uranyl acetate. Samples were dehydrated in an aqueous ethanol series (10 min in 30%, 50%, 70%, 90%, 96%, and four times in 100%, 5 min each) and rinsed twice with propylene oxide (5 min each). Samples were then left overnight in a 1:1 mixture of propylene oxide and Epon’s resin (EMBed-812 based on EPON-812). The next morning the cells were transferred to Epon and three changes (1 h each) were made before they were polymerized at 60 °C overnight. Ultrathin sections of embedded samples were made with a Leica Ultracut UCT microtome (Wetzlar, Germany), using a diamond knife. Sections were mounted on copper grids coated with Formvar film and some of the samples were stained with uranyl acetate (saturated solution in 50% ethanol) and lead citrate (saturated solution in 0.1 M NaOH). All chemicals were obtained from Sigma-Aldrich (St. Louise, USA). Sections were viewed with a Philips CM-100 TEM (Hillsboro, Oregon, USA) at the Electron Microscopy Unit of the Department of Molecular Biosciences, University of Oslo.

### Scanning electron microscopy

Cells were fixed in 2% glutaraldehyde for 1 h and 5–10 mL of fixed cell suspensions gravity filtered onto Nuclepore filters (13 mm diameter, 2 µm pore size, volumes used depended on cell density and filter clogging). Filters were rinsed in growth medium (10 min) and subsequently in 0.1 M cacodylate (10 min). Cells were post-fixed in 1% osmium tetroxide in 0.1 M cacodylate (final concentrations). Three subsequent rinses in 0.1 M cacodylate were performed (5 min each) and the cells were dehydrated in an aqueous ethanol series (10 min in 70%, 90%, 96% and three changes in 100%, 10 min each). The filter-holders were transferred in 100% ethanol to a Critical Point Dryer (Baltec CPD 030, Balzers, Liechtenstein) and the dried filters were mounted on stubs on carbon tabs. An additional protocol was followed for some of the samples; a drop of culture was placed on a poly-L-lysin coated coverslip and fixed in the vapor of 2% osmium tetroxide and left to sink overnight in a moist chamber before they were rinsed, dehydrated and critical point dried as above. All chemicals were obtained from Sigma-Aldrich (St. Louise, USA). The coverslips were mounted on stubs, sputter coated with platinum and viewed in a Hitachi S-4800 (Pleasanton, California, USA) field-emission scanning electron microscope at the Electron Microscopy Unit of the Department of Molecular Biosciences, University of Oslo and at Microbial Culture Collection at NIES (National Institute for Environmental Studies, http://mcc.nies.go.jp), Tokyo.

### Genome size

The genome size of strains was estimated by flow cytometry. Cultures were harvested before onset of the light phase during exponential growth (we observed that the seventh day after trasnfer provided more consistent results). Nuclei were released by injection of 5–10 µL of culture into 250 µl of Nuclei Isolation Buffer (NIB), previously described in Marie *et al*.^32^, diluted to 50% concentration with distilled water. The mix of cultures and NIB was incubated at 98 °C for five minutes. *Micromonas commoda* (RCC299) was added as an internal standard (genome size = 21 Mbp). The nucleic acid specific stain SYBR Green I (Molecular Probes) was added at a final dilution of 1:5000 of the commercial solution. Samples were incubated for 15 minutes before analysis on a FACS Canto II flow cytometer (Becton Dickinson) equipped with a 488 nm excitation and the standard filter setup. The procedure was repeated twice for each strain and measurements were taken in triplicate.

### Genomic DNA extraction, PCR amplification and Cloning

Cells were harvested in exponential growth phase and concentrated by centrifugation. Total nucleic acids were extracted using the Nucleospin Plant II kit (Macherey-Nagel, Düren, DE) following the manufacturer’s instructions. The nearly full length nuclear 18S rRNA gene^33^, the nuclear region containing the Internal Transcribed Spacers (ITS) 1 and 2, as well as the 5.8S rRNA gene^34^ and partial plastid 16S rRNA gene^35–37^ were obtained by PCR amplification using universal primers (Supplementary Table 1).

PCR products for 18S and plastid 16S rRNA were purified with the QIAquick PCR purification kit (QIAGEN, Hilden, Germany) and directly sequenced either at the Roscoff Biological Station Genomer platform as described below or sent to the Macrogen Company (Korea). ITS gene amplicons were cloned into PCR4-TOPO vectors (Invitrogen, Carlsbad, CA, USA) and transformed into *Escherichia coli* competent cells following the manufacturer’s instructions before sequencing. An average of ten clone inserts per strain were then amplified using M13 vector primers and purified using Exosap (USB products, Santa Clara, CA, USA). The sequences were determined using Big Dye Terminator V3.1 (Applied Biosystems) and T3 forward and T7 reverse vector primers. DNA was sequenced using an ABI prism 3100 sequencer (Applied Biosystems). Sequences have been deposited to GenBank under the following accession numbers: MF077471 - MF077519.

### ITS2 secondary structures

Forty-two ITS2 sequences (second internal transcribed spacer, separating the 5.8S and 28S rRNA genes) were obtained from the strains listed in Table 1. The ITS2 boundaries (5.8 and 28S rRNA flanking regions) were annotated using Hidden Markov Models (HMMs) and a Viridiplantae database^38^ as implemented in the ITS2 database annotation tool with the default parameters (http://its2.bioapps.biozentrum.uni-wuerzburg.de/)^39^. The partial B9 helix formed by the hybridization of 5.8 and 28S rRNA ITS2 flanking regions was checked for structural motifs known to be required for the precise removal of ITS2 during ribosomal RNA processing^25^. RNA secondary structure predictions were performed using the Mfold web interface^40^ under the default options with the folding temperature fixed at 37 °C, resulting in multiple alternative folding patterns per sequence. The preliminary structure for each sequence was chosen based on the presence of previously defined ITS2 hallmarks defined by Coleman^21,22,41,42^ and similarities among the other structures found within and between the clades. This occasionally coincided with the minimum free energy configuration. Exported secondary structures in Vienna format and the respective nucleotide sequences were aligned and visualized using 4SALE version 1.7^43,44^, and manually edited through extensive comparative analysis of each position (nucleotide) in sequences from the same clade, between clades and finally between lineages of prasinophyte clade VII. The hallmarks proposed by Caisová^45^ were used to unambiguously set the helices. The resulting consensus intramolecular folding pattern (secondary structure) for Choropicophyceae was drawn using CorelDRAW × 7. The proposed ITS2 folding pattern included: nucleotides conserved at 70% and 60% in lineages A and B, clades and branches, 100% conserved nucleotides within lineages A and B and each separate lineage. Regions without length and base pair conservation, for example the apical part of helices I and II as well as the lateral helix IIIa, were also represented. Putative CBC type changes were identified by pairwise comparison of the sequences in the conserved regions of the helices I, II and III within each clade and between clades. All changes, including hCBCs and non-CBC (e.g. N – N ↔ N × N) in all helices and positions analyzed and the positions of each nucleotide pair in the alignment are provided in Supplementary Table 5. The final alignment with the secondary structures in Vienna format is available as Supplementary Material 1.

### Phylogenetic analyses (ITS2 and concatenated 18S/Plastid 16S rRNA)

Nuclear 18S rRNA and partial plastid 16S RNA sequences obtained from the strains listed in Table 1 as well GenBank sequences belonging to members of the core chlorophytes and streptophytes were concatenated using Geneious 10.0.5^46^. Streptophytes sequences were used as outgroup. Accession numbers are provided on the phylogenetic trees. The concatenated sequences were aligned with MAFFT using the E-INS-i algorithm^47^. For ITS2, only the sequences from Chloropicophyceae strains were aligned with MAFFT using the G-INS-i algorithm^47^. For each sequence within the alignment, the preliminary secondary structure annotated in dot-bracket format was associated, generating a Vienna file which was imported to 4SALE^43,44^. The final alignment was edited on the basis of conserved secondary structures. Phylogenetic reconstructions were performed with two different methods: maximum likelihood (ML) and Bayesian analyses. The substitution models TN93 + G + I and K2 + G were selected for concatenated 18S rRNA/plastid 16S rRNA and ITS2 sequence datasets respectively, based on Akaike information criterion (AIC) and the Bayesian information criterion (BIC) options implemented in MEGA 6.06^48^. ML analysis was performed in PhyML 3.0^49^ with SPR (Subtree Pruning and Regrafting) tree topology search operations and the approximate likelihood ratio test with the Shimodaira-Hasegawa-like procedure. Markov chain Monte Carlo iterations were conducted for 1,000,000 generations sampling every 100 generations with burning length 100,000 using MrBayes 3.2.2^50^. MAFFT and MrBayes programs were run within Geneious 10.0.5^46^. Clade nodes were considered as well supported when SH-like support values and Bayesian posterior probabilities were higher than 0.7 and equal to 1.0, respectively.

Intra- and inter-clade sequence distances (*p*-distance) were calculated with combined nuclear and plastid datasets as well as for ITS2. The analysis was conducted using MEGA v. 6^48^ and all positions containing gaps and missing data were removed. All alignments are available as Supplementary Material 2.

### Multigene phylogeny

Forty five transcriptomes (Supplementary Table 2) from the Moore Foundation Marine Microbiology Transcriptome Sequencing Program (MMETSP)^51^ were selected to determine the phylogenetic placement of Chloropicophyceae based on a multigene alignment. Reads were downloaded from the MMETSP archive (http://data.imicrobe.us/project/view/104) to the ABiMS platform in Roscoff (http://abims.sb-roscoff.fr). The quality of the reads was checked by FastQC v.0.52 (http://www.bioinformatics.babraham.ac.uk/projects/fastqc/). Reads were separated into ribosomal and non-ribosomal sequences with RiboPicker v.0.4.3^52^ using as a reference the Small Subunit RNA database (SSR database) from the SILVA project (release 119)^53^. Ribosomal and non-ribosomal sequences were assembled separately using the *de novo* reconstruction method of Trinity release r20140717^54^ using default parameters. Contig abundance was estimated based on Fragments Per Kilobase Of Exon Per Million Fragments Mapped^55^ using RSEM v.1.1.17^56^. We only retained non-ribosomal contigs for which FPKM ≥ 2000 and percent of isoform ≥ 1.

Non-ribosomal contigs were analyzed by the Core Eukaryotic Genes Mapping Approach - CEGMA v.2^57^ using the Iplant Collaborative platform^58^. Using KOGs (Cluster of eukaryotic genes), CEGMA identifies a set of 458 core proteins that are highly conserved and present in a large number of taxa^59^. We selected 127 genes which were present in all transcriptomes (Supplementary Table 3) and included the *Arabidopsis thaliana* genome as a reference. Nucleic sequences were translated to amino acids and concatenated. The set of sequences was aligned with MAFFT yielding an alignment with 30,548 amino acid positions. The alignment was trimmed with Gblocks^60^ using the default parameters resulting in an alignment with 22,073 positions without gaps (Supplementary Material 3). The most appropriate model of protein evolution was determined with ProtTest v.3.2^61^ using the Akaike Information Criterion to be LG + I + G + F. Two different methods were used for phylogenetic inferences: Maximum Likelihood using PhyML 3.0^49^ and Bayesian using MrBayes v.3.2.2^50,62^. 100 bootstrap replicates were set for ML and 500,000 generations for Bayesian analysis. Convergence was checked with Tracer (http://tree.bio.ed.ac.uk/software/tracer/) and all effective sample sizes (ESS) were in excess of 350.

## Results and Discussion

### Analysis of morphology and genome size of members of prasinophyte clade VII reveals few discriminating characters between clades

Large genetic divergences, such as those observed between clades and lineages of prasinophyte clade VII^9^, are commonly associated with morphological variations. A large set of culture strains of members of prasinophyte clade VII (Table 1) were grown under identical culture conditions and morphological and genome size analyses were performed to determine whether patterns characteristic of lineages or clades exist.

**Figure 1:**
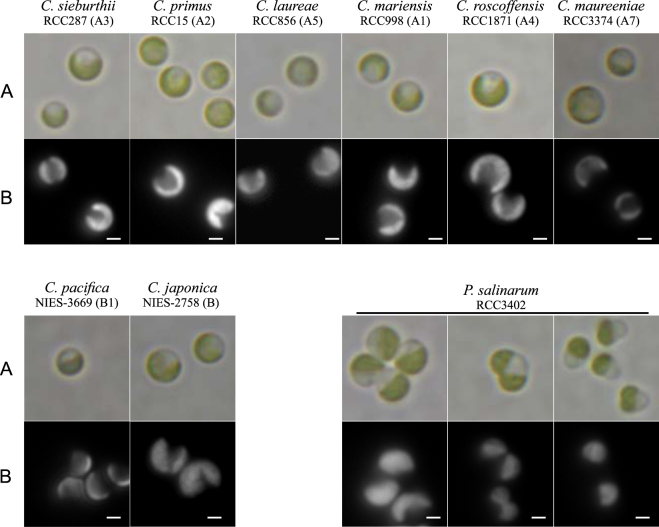
Light and fluorescence micrographs of *Chloropicon sieburthii* (A3), *C. primus* (A2), *C. laureae* (A5), *C. mariensis* (A1), *C. roscoffensis* (A4), *C. maureeniae* (A7), *Chloroparvula japonica* (NIES-2758), *C. pacifica* (B1, NIES-3669) and *Picocystis salinarum* (RCC3402). (**A**) Bright field micrographs showing cell outline, shape of chloroplasts and their color. (**B**) Black and white micrographs showing chlorophyll auto fluorescence of live cells. Scale bar: 1 µm.

In cultures, cells belonging to lineages A and B usually occur as solitary coccoid green cells, about 1.5–3 µm in size (Fig. 1 and Supplementary Figure 1A). At high cell densities, cells seem to secrete a substance that enables them to stick together and form loose colonies or aggregates (data not shown). *Picocystis salinarum* (lineage C) cells have a very different morphology. They do occur as coccoid green cells but two additional morphologies are often observed in culture: ovoid and tri-lobed (Fig. 1). As described by Lewin *et al*.^5^, these two distinct morphologies are often observed in old, nutrient-depleted cultures and their average cell size is about 2.5 µm (Supplementary Figure 1A). Transmission electron microcopy images clearly confirmed the observations of Lewin *et al*.^5^, and illustrated with more detail the bilobed chloroplast and the nucleus and single mitochondrion that occupies the third lobe of the cell in tri-lobed cells (Supplementary Figure 1).

**Figure 2:**
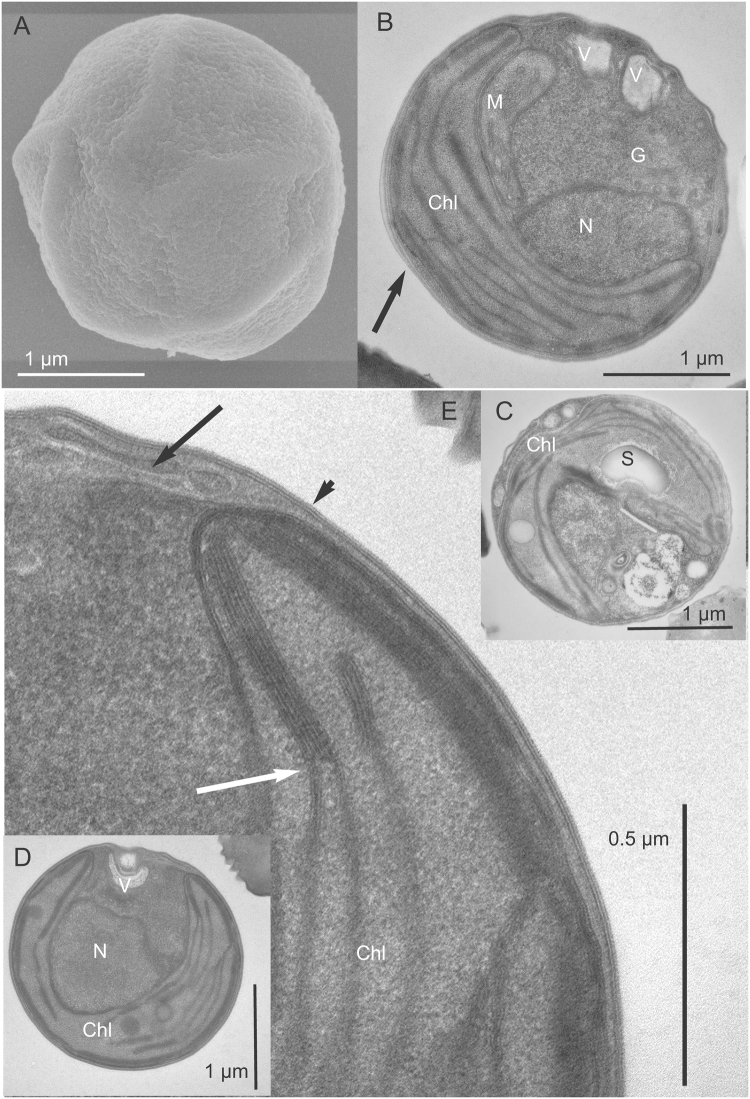
*Chloropicon sieburthii* (RCC287, A3), type species. TEM-graphs of thin sections and SEM-graphs. (**A**) Single whole cell with smooth, slightly irregular surface. (**B**) Section showing chloroplast (Chl), mitochondrion, nucleus (N), vacuoles (V), an inconspicuous Golgi apparatus and cell wall (arrow). (**C**) Starch grain (S) in chloroplast. (**D**) Cell with chloroplast showing the organization of lamella. (**E**) Enlarged part of cell showing chloroplast with lamella consisting of 1 to 4 thylakoids (white arrow) and tri-layered cell wall. The inner layer may contain inclusions (arrow).

**Figure 3:**
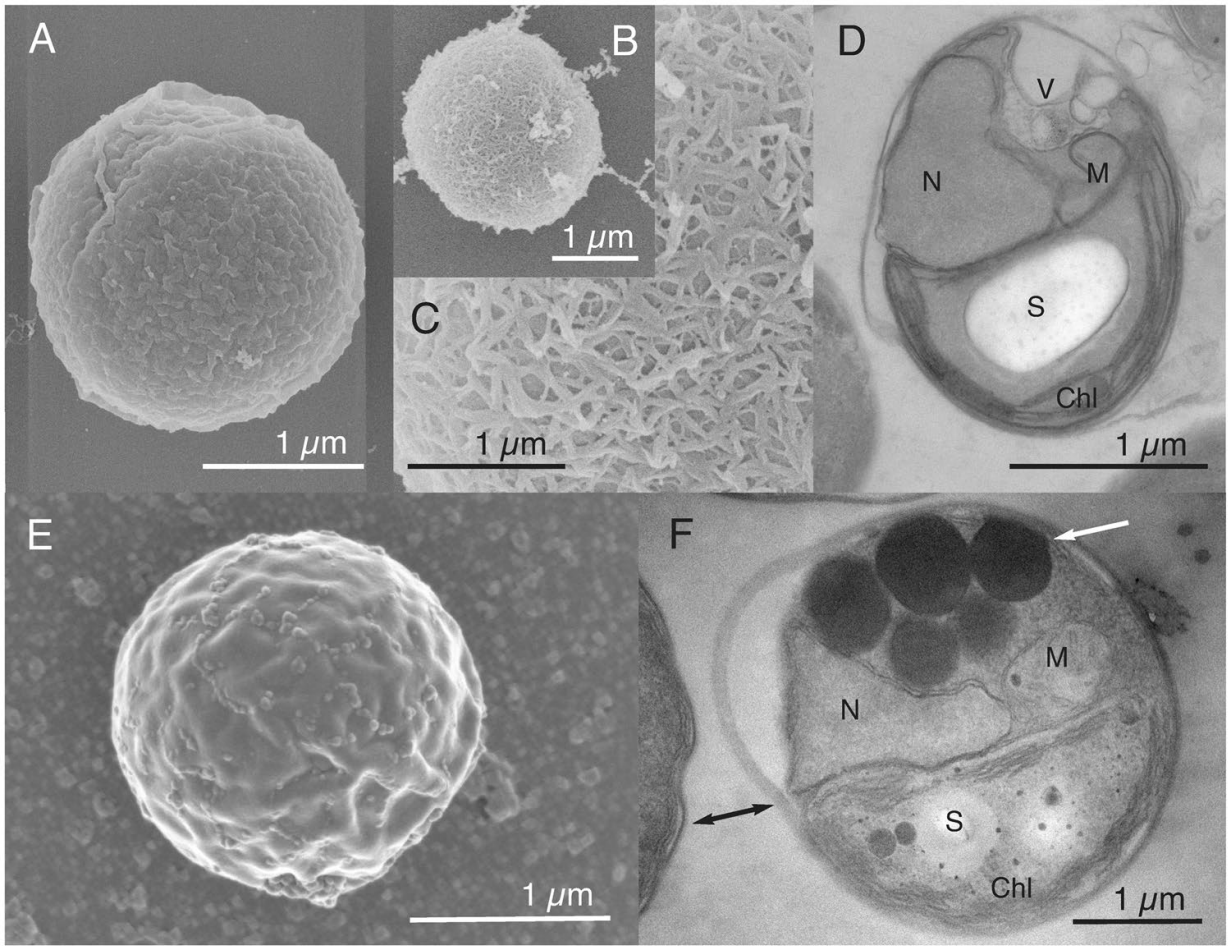
TEM-graphs of thin sections and SEM-graphs. (**A**,**B**) *Chloropicon primus* (RCC15, A2), (**C**,**D**) *C*. *laureae* (RCC856, A5). (**E**,**F**) *C*. *mariensis* (RCC998, A1), (**G**,**H**) *C*. *roscoffensis* (RCC1871, A4), (**I**,**J**) *C*. *maureeniae* (RCC3374, A7).

**Figure 4:**
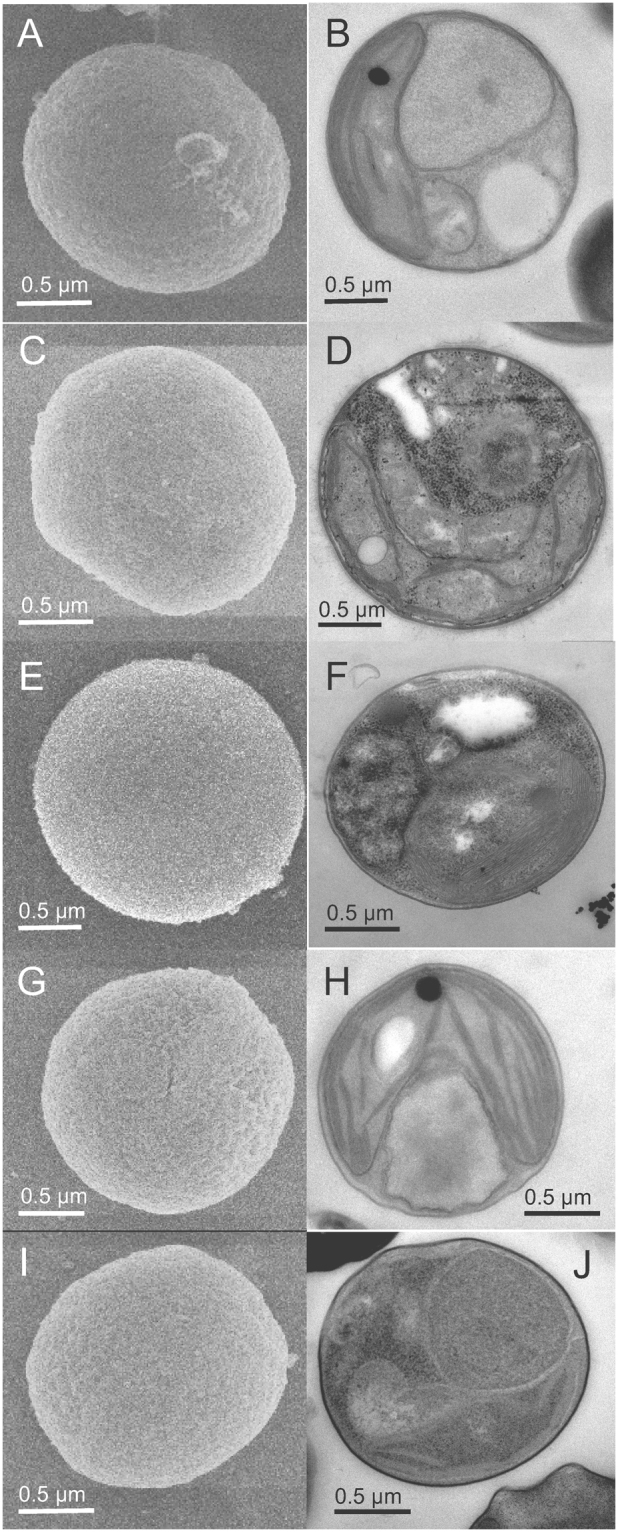
TEM-graphs of thin sections and SEM-graphs. (**A**–**D**) *Chloroparvula japonica* (NIES-2758). (**E**,**F**) *C*. *pacifica* (type species, NIES-3669, B1).

Among the strains from lineages A and B, small differences in cell size were observed between cells from the same strain or from different clades (Supplementary Figure 1A), but these differences were not consistent enough to use size as an appropriate criterion to delineate clades. Transmission and scanning electron microcopy images were also obtained for 5 strains belonging to different clades within lineage A (A1, A2, A3, A4, A5 and A7) (Figs 2 and 3) and 2 strains from lineage B (NIES-2758 and NIES-3669 from clade B1) (Fig. 4). Cells from lineages A and B normally contain one chloroplast that is often crescent shaped and harbors a starch grain (Figs 2 and 4). In dividing cells, two chloroplasts are observed (not shown). Thylakoids are commonly arranged singly or in stacks of three (Fig. 2E). The mitochondrion is located between the nucleus and chloroplast (Figs 2B and 4D and F). The Golgi apparatus seems inconspicuous and is observed only in a few sections (Fig. 2B). The cell wall is delicate and composed of layers (Fig. 2B and E). Opposite the chloroplast, vacuoles containing particles showing Brownian movement can be observed under light microscopy (Figs 2B and D and 4D). The only notable difference between the cells from lineages A and B is the fibrous cell wall (Fig. 4C), the bigger size of the starch grain (Fig. 4D) and the presence of impregnate granules in the cytoplasm (Fig. 4F), however these features were not always present.

Genome size estimated by flow cytometry ranged from 20 to almost 70 Mbp (Supplementary Figure 1B), which is higher than for picoplanktonic oceanic green algae such as *Bathycoccus*, *Micromonas* and *Ostreococcus* for which genome size is around 20 Mbp^63–65^. Karyotype analysis has not been performed for the different strains of clade VII thus the total number of chromosomes remains unknown. There are no clear differences between lineages A and B or among clades. Estimated genome size also varied between strains from the same genetic clade. These differences were particularly marked among the strains belonging to clades A4 and B2 (Supplementary Figure 1B). Strains from clade A4 formed a group with “low genome size” (RCC722, RCC726 and NIES-2755) and a group with “high genome size” (RCC917, RCC1124, RCC1871, RCC3376, RCC4429 and RCC4430) (Supplementary Figure 1B). Two strains from the “low genome size” group (RCC722 and NIES-2755) also had the smallest average cell size of all strains analyzed (Supplementary Figure 1A). The lower estimates of genome size could be the result of incomplete isolation of cell nuclei due to different composition of the cell wall in these isolates or a different level of DNA condensation. Alternatively, it has been shown for *Ostreococcus* that the size of at least two chromosomes can vary between individuals from same clade (D), which ultimately influences the DNA content of a given cell^66^. However, the global pattern of genome size was species-specific^66^. Another possibility would be that some strains have undergone diploidy as previously observed in macroalgae^67^ although this does not seem to have been observed in microalgae. Despite these differences in genome size, concatenated 18S and 16S rRNA sequence divergence within clade A4 was very low (0.1%, one substitution in 1579 analyzed positions), and ITS2 sequences were identical for all strains (see below). In contrast, B2 showed the highest intra-clade sequence divergence for both the combined dataset and ITS2 sequences (see below) which may correspond with the differences in estimated genome size.

### Pigment composition differs among the different lineages and clades of prasinophyte clade VII

Pigment signature has traditionally been applied as a taxonomic proxy of algal diversity in oceanography^68^. Pigment composition can be closely connected to environmental adaption^69^. The pigment composition of prasinophyte clade VII strains belonging to lineages A and B is typical of green algae (Chlorophyta). They all contain the following set of carotenoids: neoxanthin, violaxanthin, antheraxanthin, zeaxanthin, lutein and β,ε – carotene^8,70^. The pigment composition of lineage C, *Picocystis salinarum*, is unusual in that it contains alloxanthin and monadoxanthin (typical of cryptophytes^71^) and diatoxanthin (typical of heterokonts^68^) in addition to chlorophyll *a* and *b* and the basic set of carotenoids commonly found in green algae^5,8^. Alloxanthin and diatoxanthin have also been reported in *Coccomyxa*–like algae^72^, a chlorophyceaen pathogen of the mussel *Mytilus galloprovincialis*.

In the present study, we provide pigment data for 7 additional strains from lineages A and B, meaning that pigment signatures are available for 21 strains (Table 2). Violaxanthin and lutein were the two most abundant carotenoids for most of the strains from lineages A and B, astaxanthin coming third when it is present. Astaxanthin has previously been shown to increase with light intensity in prasinophyte clade VII (from 2- to 4-fold depending on the strain), suggesting a photoprotective role for this carotenoid^8^. Of the 21 strains, only NIES-3669 belonging to clade B1 did not possess astaxanthin and β,β – carotene as accessory carotenoids. In contrast, this strain had an antheraxanthin to Chl *a* ratio 10 times higher than that of the other strains (Table 2). Antheraxanthin is part of the photoprotective epoxidation and de-epoxidation cycle VAZ (violaxanthin-antheraxanthin-zeaxanthin) and its content can be variable depending on light conditions^73^.

Loroxanthin was also detected among some strains from lineages A and B (Table 2) and has previously been suggested to have a major light harvesting role since it increases at low light intensity^8^. Loroxanthin was absent in clade B1 (NIES-3669), B3 (RCC4572), in one strain from clade B2 (RCC696) and clade A4 (Table 2). Several strains from clade A4 were isolated from northern or southern temperate latitudes (~49° N and ~33° S) or from tropical regions (Table 1). The two clade A4 strains previously analyzed (RCC1124 and RCC1871), isolated from temperate North Atlantic Ocean waters, lacked loroxanthin. In the present study five other clade A4 strains from a wider range of latitudes were analyzed. The data confirm that all strains of clade A4, whatever the latitude from which they were isolated, lacked loroxanthin (Table 2). Clearly the absence of loroxanthin is a phenotypic characteristic of clade A4, but it is also absent from most strains from lineage B, and therefore cannot be used as a biomarker to distinguish A4 from all other members of prasinophyte clade VII.

Phylogenomic analysis of chloroplast sequences have suggested that prasinophyte clade VII lineage A is a sister group of the core Chlorophyta^12,74,75^. The core Chlorophyta comprise a well-supported clade containing the classes Chlorophyceae, Ulvophyceae, Trebouxiophyceae, Chlorodendrophyceae and Pedinophyceae. The presence of astaxanthin and loroxanthin carotenoids in clade VII lineage A and B strains, as in core Chlorophyta, while it is absent in other prasinophytes is indicative of another common feature between this group of prasinophytes and core Chlorophyta.

### Nuclear and plastid SSU rRNA as well as ITS2 phylogeny support clade separation

Our previously published phylogenetic analyses based on nuclear and plastid SSU rRNA gene sequences demonstrated that lineage C did not contain any sub-division. In contrast, lineages A and B could be further divided intoclades: A1 to A7 and B1 to B3^9^. Each clade was defined based on the presence of at least two environmental or strain sequences obtained from different locations (and/or samples at a given location) with strong phylogenetic support for at least one of the gene markers used. Clade B3 was composed only by environmental sequences^9^. Strain RCC4572, recently isolated from the Atlantic Ocean^76^, had signatures for both nuclear and plastid SSU rRNA genes previously identified as clade B3. This means that all clades known from the environment^9^ have now been brought into culture.

**Figure 5:**
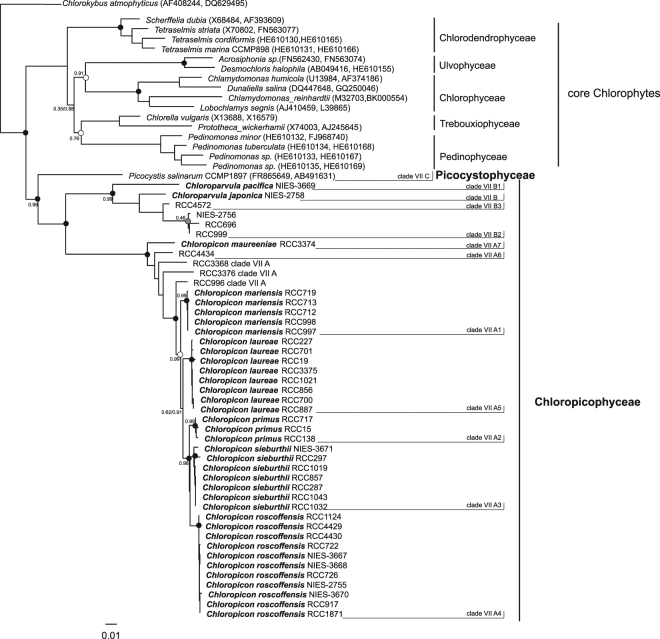
Maximum-likelihood tree inferred from concatenated plastid and nuclear sequences of prasinophytes clade VII. Sequences belonging to members of the core Chlorophytes and Streptophytes were included in addition to the sequences obtained from the cultures. Streptophytes were used as an outgroup. Solid dots correspond to significant support (>0.7) for ML analysis and full support (1.0) by Bayesian analysis. When ML support is below 1.0 the percentage is indicated next to the symbol. Grey dots correspond to non-significant ML support (<0.7) and full support from Bayesian analysis. Empty dot corresponds to ML support but no support from Bayesian analysis.

A phylogenetic analysis combining partial plastid SSU rRNA gene sequences with a congruent data set of nuclear 18S rDNA sequences (Fig. 5) recovered the major diverging clades within prasinophyte clade VII lineages A and B with high support values for maximum likelihood (ML) and Bayesian analyses. The only exception was clade B2 which had no support from ML (0.46) analysis (Fig. 5). Phylogenetic analysis based on ITS2 (internal transcribed spacer 2) sequences from 41 unique strains from lineages A and B also confirmed the major divergent clades described above (Supplementary Figure 3). However, both analyses (combined SSU rRNA and ITS2) failed to resolve the relationships between the different clades (low bootstrap and variable tree topologies).

The uncorrected sequence distance (*p*-distance) within clades calculated with the combined 18S nuclear and 16S plastid datasets was below 0.04% (for clade B2), while interclade divergence was as high as 13.5% (between A5 and B2, Supplementary Table 4). The distance observed between lineages A and B (12.6%) is similar to the divergence found for example between Chlorodendrophyceae and lineage A. The interclade sequence distance varied from 1.0% (i.e. A2 vs. A3) to 4.3% (i.e. A2 vs. A7) for A and 2.2% (i.e. B2 vs. B3) to 7.7% (i.e. B1 to B3) for B (Supplementary Table 4).

Interclade distances using ITS2 sequences were higher with a maximum value of 42% between A2 and B1. Within the clades, the distance varied from 0% in A4 to 5.6% in B2. The high sequence *p*-distance found in B2 suggests that this clade may represent different species (see below). However, these values should be taken with caution given the inherent difficulty in aligning ITS regions (Supplementary Table 4).

The genetic divergence found between clades within prasinophyte clade VII suggests that they may each represent different species. In another group of prasinophytes, *Micromonas*, similar genetic divergence values have been observed for the highly conserved 18S rRNA gene among different clades recently erected to species status^77^. For the picoplanktonic *Ostreococcus*, the highest sequence distance, also based on 18S rRNA analyses, was 1.8% between two clades that are now considered to represent different species^66^.

### ITS2 structure confirms clade separation

**Figure 6:**
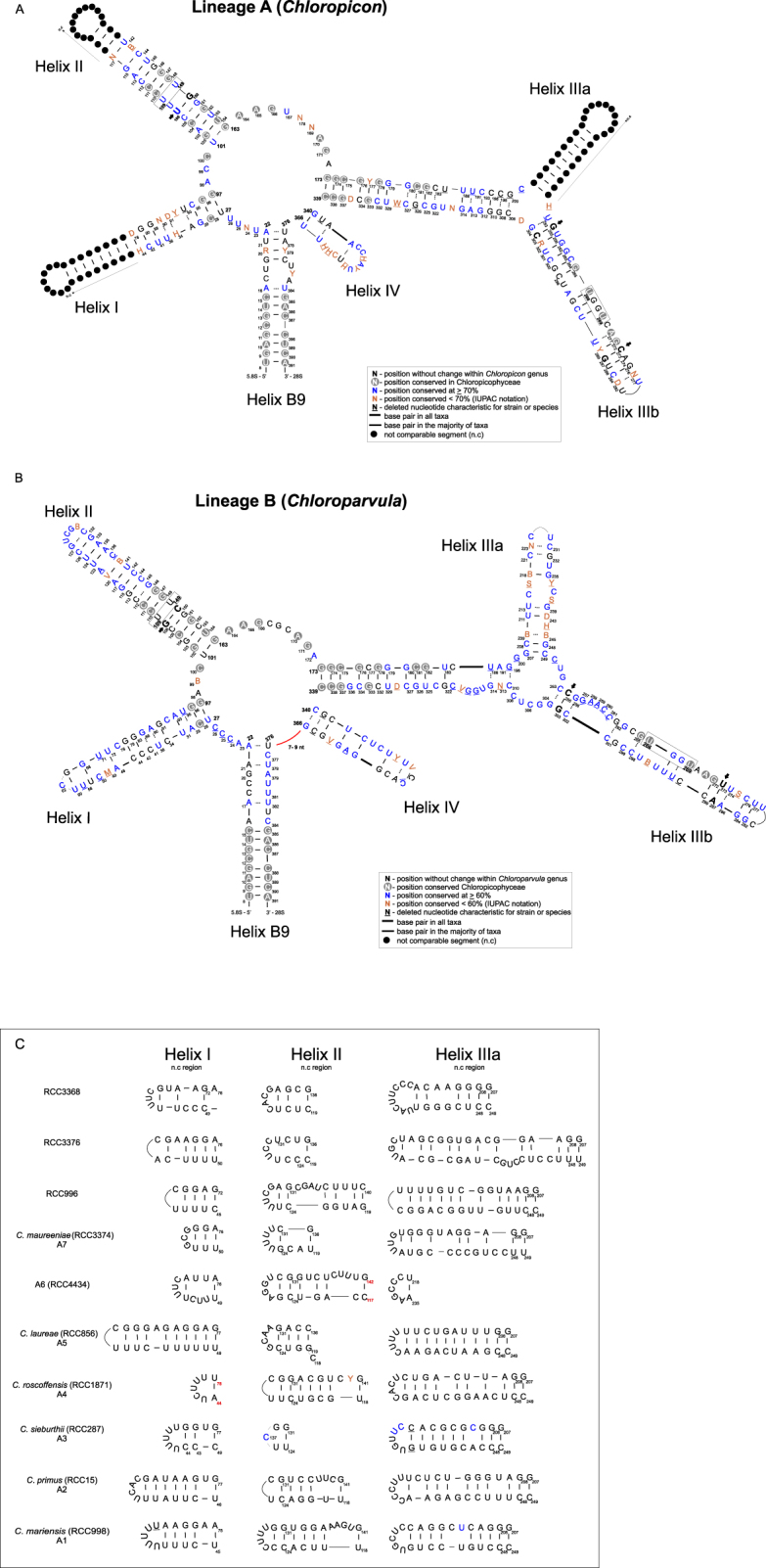
Consensus secondary structure model of the ITS2 molecule of Chloropicophyceae with the two genera, A) *Chloropicon* (lineage A) and B) *Chloroparvula* (lineage B). The four major helices are labeled as Helix I – Helix IV and the interaction region of 5.8S and 28S rRNA as B9. Nucleotide letters shown in blue in both ITS2 diagrams refer to those present in 70% (**A**) and 60% (**B**) of the clades and branches analyzed. Any position with less than the majority rule applied are shown as IUPAC ochre symbols. Invariable positions within each lineage are drawn in black and circled in grey when common to both A and B lineages. Arrows and nucleotides in bold indicate the major three CBCs between the two lineages. Positions with deletions are underlined. Regions without length and base pair conservation are shown as black dots. These regions, corresponding to the apical part of helices I and II as well as the lateral helix IIIa are drawn for each clade/branch in the panels on the right side.

In order to examine in more depth the level of inter- and intra-clade diversity of prasinophyte clade VII we analyzed the secondary structure of ITS2 for 41 strains listed in Table 1 (Fig. 6). Unfortunately, since data are only available for one strain of *Picocystis* (clade C), it is not possible to determine the ITS2 folding pattern for this lineage.

The ITS2 secondary structure of lineages A and B contained the four-helix domains known in many eukaryotic taxa in addition to helix B9 (Fig. 6). Helix B9, a region of the 5.8S and 28S rRNA interaction, shows the highly conserved eight base pair stem required for the precise excision of the ITS2^25^. Helices II and III harbor the universal hallmarks proposed by Mai and Coleman^20^ and Schultz *et al*.^78^: the pyrimidine-pyrimidine (Y-Y) mismatch in helix II and YRRY (pyrimidine – purine – pyrimidine) motif on the 5′ side of Helix III (boxes, Fig. 6). The Y-Y mismatch was U × U for all sequences analyzed, with the exception of clade A6 (C × U) and the solitary branch RCC3368 (U × C). The YRRY motif of helix III was represented by the sequence UGGU in all strains analyzed, except for NIES-2756 (clade B2) where the guanidine is replaced by adenine (UAAU). The spacers between helices I and II and between II and III displayed the fixed number of nucleotides proposed by Caisová *et al*.^45^ (Fig. 6). Spacers between helices B9 and I, III and IV and IV and B9 were less conserved when compared to the Chlorophyta consensus structure and within lineage B. Remarkably, the secondary structure of lineage B exhibited an insertion of 7–9 nucleotides between helices III and B9 which was completely absent in lineage A and NIES-2758 presented a unique deletion of 3 nucleotides within the spacer between helices B9 (Fig. 6B). Among the conserved spacers (between helices I – II and II – III), not only the length was conserved but also the nucleotides occupying the alignment positions 98 (A), 100 (C), 164 to 166 (AAG) and 170 to 172 (AGA) were conserved with respect to the ITS2 Chlorophyta consensus secondary structure (for details see Fig. 3 of Caisová *et al*.^45^) (Fig. 6). The only exceptions were clades A6 (RCC4434) with one uracil at position 98 and B1 (NIES-3669), also with a replacement of A by uracil at position 172. Since these clades were represented by a single strain, we cannot confirm whether these changes are characteristic of these clades.

The first two base pairs of helices I, II and III are another important hallmark for the unambiguous identification of these helices. They were conserved within lineages A and B (Fig. 6) and in agreement with the consensus ITS2 structure of Chlorophyta (for details see Fig. 3 of Caisová *et al*.^45^). However, the three-nucleotide motif (AGG) on the 5′ side of the base of Helix IV, also proposed by Caisová *et al*.^45^, was not detected in our structures (Fig. 6).

Two approaches have been proposed for CBC identification: 1) phenetic, whereby in base pair sequence comparison all CBCs between two sequences are considered, without direct reference to their evolutionary origin^22,26^, and 2) phylogenetic, which considers the status of a given base pair in the ancestor of two sister taxon a priori the determination of the CBC^45,79,80^. Unfortunately, the phylogenetic approach could not be used in our study given the conflicting branching pattern among phylogenies (Fig. 5, Supplementary Figure 3). Table 3 details the number of CBCs between two branches or clades and the nucleotide pair identification number where CBCs were found (numbers in brackets).

**Table 3:** CBCs in the conserved regions of the helices I, II and III within each clade and between the clades. The numbers in bold represent the number of CBCs found between two branches or clades. The numbers in brackets represent the CBC identification number (see Supplementary Table 5 for the list of all CBC and for their position on the ITS2 alignment).

	*Chloropicon mariensis* (A1)	*Chloropicon primus* (A2)	*Chloropicon sieburthii* (A3)	*Chloropicon roscoffensis* (A4)	*Chloropicon laureae* (A5)	RCC4434 (A6)	*Chloropicon maureeniae* (A7)	RCC996 (A)	RCC3368 (A)	RCC3376 (A)	RCC2337 and RCC999 (B2)	RCC696 (B2)	*Chloroparvula japonica* (NIES-2758)	*Chloroparvula pacifica* (B1)
***Chloropicon mariensis*** (**A1**)	**0**													
***Chloropicon primus*** (**A2**)	**1** [13]	**0**												
***Chloropicon sieburth****ii* (**A3**)	**4** [9, 13, 50, 57]	**2** [13, 20]	**0**											
***Chloropicon roscoffensis***(**A4**)	**2** [9, 13]	**0**	**4** [13, 20, 51, 57]	**0**										
***Chloropicon laureae*** (**A5**)	[20, 36, 50]	**4** [9, 13, 29, 36]	**5** [9, 13, 20, 36, 57]	**3** [9, 13, 20]	**0**									
**RCC4434** (**A6**)	**4** [18, 19, 20, 57]	**5** [9, 13, 18, 19, 20]	**4** [9, 18, 19, 50]	**6** [9, 13, 18, 19, 20, 57]	**5** [13, 18, 20, 50, 57]	**0**								
***Chloropicon maureeniae*** (**A7**)	**5** [9, 10, 14, 15, 57]	**5** [10, 13, 14, 15, 48]	**8** [10, 14, 15, 20, 50, 51, 57, 58]	**5** [10, 13, 14, 15, 57]	**8** [9, 10, 13, 14, 15, 20, 50, 57]	**9** [9, 10, 14, 15, 18, 19, 20, 57, 58]	**0**							
**RCC996** (**A**)	**0**	**2** [10, 13]	**4** [10, 13, 20, 57]	**1** [13]	**4** [10, 20, 36, 50]	**6** [10, 13, 18, 19, 20, 57]	**5** [10, 13, 14, 15, 57]	**0**						
**RCC3368** (**A**)	**5** [9, 10, 13, 30, 57]	**3** [9, 10, 36]	**8** [9, 10, 13, 20, 30, 36, 50, 57]	**4** [9, 10, 30, 57]	**4** [9, 10, 30, 57]	**7** [9, 10, 13, 18, 19, 20, 57]	**5** [9, 13, 14, 15, 58]	**5** [10, 13, 30, 36, 57]	**0**					
**RCC3376** (**A**)	**3** [19, 34, 35]	**4** [9, 13, 19, 35]	**6** [9, 19, 20, 35, 51, 57]	**4** [9, 13, 19, 35]	**4** [19, 20, 35, 36]	**4** [18, 20, 50, 57]	**6** [9, 14, 19, 35, 50, 57]	**4** [13, 19, 35, 50, 51]	**7** [9, 13, 19, 35, 36, 50, 57]	**0**				
**RCC2337 and RCC999** (**B2**)	**14** [8, 9, 14, 16, 18, 35, 40, 41, 49, 54, 55, 56, 57, 58]	**16** [7, 8, 13, 14, 16, 18, 35, 37, 40, 41, 48, 50, 54, 55, 56, 58]	**15** [8, 14, 16, 18, 20, 35, 37, 40, 41, 49, 50, 54, 55, 56, 58]	**17** [7, 8, 13, 14, 16, 18, 35, 40, 41, 49, 50, 51, 54, 55, 56, 57, 58]	**10** [8, 9, 11, 13, 14, 54, 55, 56, 57, 58]	**14** [7, 9, 14, 16, 18, 19, 20, 37, 49, 50, 54, 55, 56, 58]	**16** [7, 8, 10, 15, 16, 18, 35, 40, 41, 49, 50, 51, 54, 55, 56, 58]	**18** [7, 8, 10, 13, 14, 16, 18, 33, 35, 40, 41, 49, 50]	**18** [7, 8, 9, 10, 13, 14, 16, 18, 30, 35, 40, 41, 49, 50, 54, 55, 56, 58]	**16** [7, 8, 14, 16, 18, 19, 40, 41, 49, 50, 51, 54, 55, 56, 57, 58]	**0**			
**RCC696** (**B2**)	**12** [8, 9, 14, 16, 18, 40, 41, 49, 54, 55, 56, 57]	**13** [7, 8, 13, 14, 16, 18, 37, 40, 41, 49, 54, 55, 56]	**12** [8,14, 16, 18, 20, 37, 40, 41, 49, 54, 55, 56]	**15** [7, 8, 13, 14, 16, 18, 37, 40, 41, 49, 51, 54, 55, 56, 57]	**15** [7, 8, 9, 11, 13, 14, 16, 18, 20, 41, 49, 54, 55, 56, 57]	**10** [9, 14, 16, 18, 19, 20, 49, 54, 55, 56]	**11** [7, 8, 10, 15, 16, 18, 40, 51, 54, 55, 56]	**11** [7, 8, 10, 13, 14, 16, 18, 41, 54, 55, 57]	**17** [4, 7, 8, 9, 10, 13, 14, 16, 18, 30, 37, 40, 41, 49, 54, 55, 56]	**15** [7, 8, 14, 16, 18, 19, 37, 40, 41, 49, 51, 54, 55, 56, 57]	**7** [21, 41, 42, 43, 44, 45, 47]	**0**		
***Chloroparvula*** ***japonica*** (**NIES-2758**)	**11** [8, 9, 14, 16, 18, 20, 40, 41, 49, 50, 54]	**12** [8, 13, 14, 18, 40, 41, 48, 49, 50, 54, 58]	**12** [8, 14, 16, 20, 40, 41, 49, 50, 54, 57, 58]	**12** [8, 13, 14, 16, 18, 40, 41, 49, 50, 51, 54, 58]	**16** [8, 9, 13, 14, 16, 18, 20, 27, 29, 40, 41, 48, 49, 50, 54, 58]	**11** [9, 14, 16, 18, 19, 20, 49, 50, 54, 57, 58]	**12** [8, 15, 16, 18, 40, 41, 49, 50, 51, 54, 57, 58]	**10** [4, 8, 13,14, 16, 18, 49, 50, 54, 58]	**15** [8, 9, 13, 14, 16, 18, 30, 40, 41, 48, 49, 50, 54, 57, 58]	**13** [8, 9, 14, 16, 18, 19, 40, 41, 49, 50, 51, 54, 58]	**8** [20, 21, 22, 23, 42, 43, 56, 57]	**10** [20, 21, 22, 23, 24, 41, 43, 45, 56, 57]	**0**	
***Chloroparvula pacifica*** (**B1**)	**12** [9, 14, 16, 19, 20, 29, 30, 40, 41, 49, 50, 54]	**9** [13, 14, 16, 19, 29, 40, 41, 49, 54]	**11** [14, 16, 19, 20, 29, 30, 40, 41, 49, 54, 57]	**9** [13, 14, 10, 19, 29, 30, 49, 54, 58]	**11** [9, 13, 14, 16, 19, 20, 29, 30, 40, 49, 54]	**11** [9, 14, 16, 19, 20, 29, 30, 49, 50, 54, 57]	**10** [15, 16, 19, 29, 30, 49, 50, 54, 57, 58]	**13** [8, 13, 14, 16, 18, 49, 50, 54, 58, 40, 41, 49, 50, 54]	**12** [9, 13, 14, 16, 19, 29, 30, 40, 49, 50, 54, 57]	**9** [9, 14, 16, 29, 30, 40, 41, 49, 54]	**15** [8, 20, 21, 22, 23, 24, 25, 29, 30, 37, 40, 45, 50, 56, 57, 58]	**15** [8, 19, 20, 21, 22, 23, 24, 25, 29, 30, 37, 40, 41, 56, 57]	**10** [8, 19, 21, 24, 29, 30, 40, 45, 50, 58]	**0**

Caisová *et al*., using a phylogenetic approach, showed that in two classes of green algae, Ulvales^79^ and Chlorophyceae^45^, CBCs on Helix II and III were often correlated with divergences at supra-specific taxonomic levels, for example genus. A significant number of CBCs, mainly localized in helices II and III, were observed between clades belonging to different lineages (Table 3). Three CBCs (bp position 16, 49 and 54, Supplementary Table 5) in helices II and III distinguished lineage A from B (Fig. 6).

Within clades, only clade B2 had CBCs (and hCBCs) in helices II and III, between strain RCC696 on the one hand and the other two, RCC999 and NIES-2756, on the other (Table 3). In fact, sequence divergence within this clade was highest among the clades for both sequence datasets used (Supplementary Table 4) and the pigment composition of RCC696 was slightly different from that of NIES-2756 since it lacked loroxanthin (Table 2). Within the other clades of lineages A and B, the ITS2 sequences were nearly identical and neither CBCs nor hCBCs were detected (Table 2, Supplementary Table 5).

In general, several CBCs were detected between clades or solitary branches. There were two exceptions for which CBCs were not observed: between RCC996 and A1 and between A4 and A2 (Table 3), although hCBCs (7 and 6 respectively) were found in helices I, II and III (Supplementary Table 5). All clades and branches, including these ones, can be differentiated by molecular signatures present in both plastid and nuclear SSU rRNA genes, a fact that was further confirmed here. The branch formed by RCC996 has not been classified within a specific clade^9^ given the absence of similar complete 18S rRNA gene sequence from other cultures or environmental samples. In addition, A1 possesses a 500 bp long 18S rRNA intron around position 1,000 which is not detected in any other clade. While few sequences corresponding to that of RCC996 have been found in metabarcoding datasets^9^, clade A1 was relatively more important at the DCM (deep chlorophyll maximum) of some Pacific *Tara* Ocean stations fitting the fact that all A1 strains have been isolated from deep euphotic waters^9^. Another example of distinct molecular signatures congruent with ecological specificities was provided by contrasting clades A4 and A2. Clade A4 is the second most abundant clade after B1 and is mainly found in coastal waters (OSD dataset), probably indicating a habitat preference, while the abundance and distribution of A2 is very sporadic^9^. Strains belonging to A4 differed from others by the absence of loroxanthin (Table 2). Thus, despite the absence of CBCs between clade A1 and RCC996 or between A2 and A4, each probably represents organisms with distinct biological and ecological properties.

### Nuclear multigene phylogeny confirms the monophyly of lineages A and B

**Figure 7:**
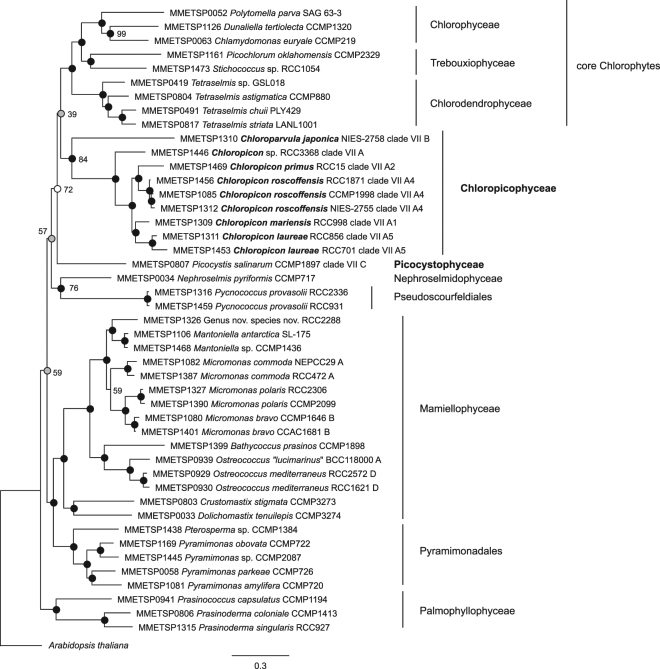
Maximum Likelihood (ML) phylogenetic tree based on a concatenated alignment of 22,073 amino acids corresponding to 127 nuclear core genes extracted from transcriptomes obtained for 45 Chlorophyta strains obtained in the framework of the Marine Microbiology Initiative (MMETSP)^51^. Solid dots correspond to significant support (>70%) for ML analysis and full support (100% probability) by Bayesian analysis. When ML support is below 100%, the percentage is indicated next to the symbol. Grey dots correspond to non-significant ML support (<70%) and full support from Bayesian analysis. Empty dot corresponds to ML support but no support from Bayesian analysis.

A multigene phylogenetic analysis was performed using the transcriptome sequence database obtained in the frame of the MMETSP Marine Microbiology Initiative^51^. Forty-five transcriptomes were selected including all those available for prasinophyte clade VII as well as from related Chlorophyta lineages (Supplementary Table 2). The Core Eukaryotic Genes Mapping Approach (CEGMA) defines a set of 458 core nuclear genes for which HMM profiles are available. Of these, 127 genes (Supplementary Table 3) were found in all of the selected transcriptomes and these were used to establish a multigene phylogeny based on a concatenated amino acid alignment of 22073 positions. In this analysis, the transcriptomes from 9 strains belonging to lineages A and B formed a moderately supported clade, independent of lineage C, confirming their monophyly (Fig. 7). This clade was a sister clade to core Chlorophyta, although with low ML support in contrast to the strong support reported by Lemieux *et al*.^12^ for a phylogenomic analysis based on chloroplast sequences. The position of lineage C (*Picocystis salinarum*) varied depending on the method used. In the ML analysis, *Picocystis* formed a branch of its own, weakly related to prasinophyte clade VII and core chlorophytes (Fig. 7). In previous chloroplast genome phylogenies *Picocystis* branched with Pseudoscourfeldiales^75^ or formed an independent branch^12^.

Within lineage A, the major clades for which transcriptomes were available (A1, A2, A4 and A5) and solitary branches defined by strains NIES-2758 and RCC3368 (CCMP2111) were recovered in the multigene analysis (Fig. 7). The latter strains cannot be ascribed to any of the clades previously defined^9^ because no other culture or environmental sequences are similar to them. In accordance with 16S/18S and ITS phylogenies (Fig. 5, Supplementary Figure 3), clades A2 and A4 were closely related in the multigene analysis (Fig. 7). Clades A1 and A5 formed a cluster with 100% support, but this association was not observed in 18S/16S and ITS2 rRNA analyses (Fig. 5, Supplementary Figure 3). In the multigene analysis all other Chlorophyta groups (Chlorodendrophyceae, Chlorophyceae and Trebouxiophyceae which belong to the core Chlorophyta), as well as Mamiellophyceae, Pseudoscourfeldiales, Nephroselmidophyceae and Palmophyllophyceae were monophyletic with 100% bootstrap support in both methods.

### Prasinophyte clade VII comprises 2 new classes containing three genera and 8 species

A reassessment of the taxonomy of prasinophytes has been needed for some time. The idea of raising prasinophyte lineages to class status was proposed by Nakayama *et al*.^81^, based upon the paraphyletic nature of prasinophyte lineages, their genetic dissimilarity based on rRNA sequences and the recognition of well supported classes in the core Chlorophyta. The differentiation of clades proposed by Guillou *et al*.^4^ was a first step, followed by the erection of novel classes replacing some of these clades: Mamiellophyceae for clade II^10^, Chlorodendrophyceae for clade IV^82^, and Palmophyllophyceae for clade VI^75^. The phenotypic and genetic data that we obtained on a large set of culture strains in the present study allows clarification of the taxonomy of clade VII which is ecologically important in oceanic waters^9^. All of the analyses performed on strains from lineages A and B converge to establish that these two lineages share many phenotypic and genetic traits, including similar morphology (Figs 1–4), similar pigment composition (Table 2), and monophyly in all phylogenetic analyses (Figs 5 and 7 and Supplementary Figure 3). *P*. *salinarum* was originally grouped with prasinophyte clade VII based on 18S rRNA phylogenetic analyses, forming lineage C restricted to this species^4^. The degree of sequence similarity between the nuclear 18S RNA gene sequence of *P*. *salinarum* and those of other prasinophyte clade VII (around 88%) is comparable to that between lineages A and B^9^. The concatenated nuclear 18S/plastid 16S rRNA phylogenetic tree (Fig. 5) gave the same result. However, in phylogenetic analysis using only plastid 16S rRNA gene sequences, lineage C formed an independent lineage from prasinophyte clades VII A and B^9^. Moreover, phylogenetic analyses using the complete nuclear^11^, plastid encoded rRNA operons^10,11^ and chloroplast genomes^12^ already suggested that *P*. *salinarum* forms a separate lineage from prasinophyte clade VII A and B. In all of these analyses, only the data from RCC15 (CCMP1205, clade A2) and RCC3402 (CCMP1897, *P*. *salinarum*) were used. Multigene phylogeny (Fig. 7), morphology (Supplementary Figure 2) and pigment composition, in particular the presence of red lineage carotenoids^5,8^ (Table 2) provide compelling evidence that prasinophyte clade VII and *P*. *salinarum* should be considered independent lineages and that these represent distinct classes of prasinophytes. Therefore, we have raised prasinophyte clade VII lineages A and B together to class status as the Chloropicophyceae and we also create the class Picocystophyceae to accommodate the genus *Picocystis* described by Lewin^5^.

Few morphological characters distinguish Chloropicophyceae from other picoplanktonic prasinophytes. Among described green algae, there are four genera containing naked coccoid non-motile cells: *Prasinoderma*, *Picochlorum*, *Pycnococcus* and *Ostreococcus*. The pyrenoid is easily observed in *Pycnococcus*^83^ and *Prasinoderma*^84^, whereas it is absent in Chloropicophyceae (Figs 2–4). Sexual reproduction or auto-sporulation have been proposed for *Pycnococcus*^83,85^ and *Picochlorum*^86^, but have never been observed in Chloropicophyceae cells. Pigments composition is perhaps the most distinctive character between Chloropicophyceae and these four genera. *Pycnococcus*, *Prasinoderma* and *Ostreococcus* belong to pigment group prasino-3A and 3B^68^, all containing prasinoxanthin^70^, which is absent in Chloropicophyceae (Table 1) and *Picochlorum*^86^. The carotenoids astaxanthin and loroxanthin are found in cells of Chloropicophyceae, while they are absent in *Picochlorum*.

Within the Chloropicophyceae we establish two new genera: *Chloropicon* and *Chloroparvula*, corresponding to lineages A and B, respectively. Certain morphological features distinguish lineage B (*Chloroparvula*) cells from those of lineage A (*Chloropicon*): presence of a fibrous cell wall (Fig. 4C), the larger size of the starch grain (Fig. 4D) and the presence of impregnate granules in the cytoplasm (Fig. 4F). Despite overall morphological similarity, lineages A and B formed independent monophyletic lineages in our multigene phylogeny, with *Chloropicon* receiving 100% support with both methods used (Fig. 5). Unfortunately only one transcriptome was available for the genus *Chloroparvula* (previously clade VIIB), so it was not possible to assess multigene phylogeny support for the erection of this genus. The average genetic distance observed between lineages A and B (around 12%) in our concatenated dataset is similar to the divergence between well-established classes of the core Chlorophyta (Supplementary Table 4), justifying separation of these two lineages (at least) at the genus level.

Müller *et al*.^26^ and Caisová *et al*.^45,79^ showed that the absence of CBCs in ITS2 secondary structures is not an indicator that two organisms belong to the same species. This is particularly true for Ulvales and Chlorophyceae for which a lack of correlation between CBCs in ITS2 at the species level was reported^45,79^. However, the presence of at least one CBC is a good indicator that two organisms represent distinct species (93.1% confidence for plants and fungi^26^). For picoeukaryotes that are indistinct morphologically^66,77^, form complex species^80,87,88^ or are even uncultured^89^, this distinguishing character may be particularly useful. ITS2 secondary structure analyzed together with molecular signatures of nuclear and plastid SSU rRNA genes support the hypothesis that Chloropicophyceae clades and branches represent distinct species, despite the absence of clear morphological differences. In addition to knowledge on their ecological distribution, these results lead us to erect to species status 7 clades (A1, A2, A3, A4, A5, A7 and B1) and one solitary branch (NIES-2758) for which we have ultrastructural information. The other clades (A6, B2 and B3) were not erected to species level due to the absence of EM images necessary to establish holotypes. The new species of Chloropicophyceae are: *Chloropicon mariensis* (A1), *Chloropicon primus* (A2), *Chloropicon sieburthii* (A3), *Chloropicon roscoffensis* (A4), *Chloropicon laureae* (A5), *Chloropicon maureeniae* (A7), *Chloroparvula japonica* (NIES-2758) and *Chloroparvula pacifica* (B1).

The formal taxonomic description of prasinophyte clade VII as the new class Chloropicophyceae will facilitate interpretation of large-scale metabarcoding and/or metagenomics analyses that aim at investigating the ecological patterns and the role in the marine environment of this enigmatic group of oceanic picoplanktonic green algae.

## Taxonomy section

Chlorophyta Reichenbach 1834

Chloropicophyceae Lopes dos Santos and Eikrem classis nov.

Diagnosis: Coccoid green cells, with a diameter of 1.5–4 µm, found in marine waters. One nucleus, one mitochondrion, one chloroplast surrounded by two membranes, containing starch grain. Chloroplast with chlorophylls *a* and *b*. Pyrenoid absent. Flagella absent. Coccoid cells with layered cell wall. Sexual reproduction unknown.

Chloropicales Lopes dos Santos and Eikrem ord. nov.

Diagnosis: With characters of class. Additional characters; accessory pigments are neoxanthin, violaxanthin, antheraxanthin, zeaxanthin, lutein, loroxanthin, astaxanthin, β,β- carotene, β,ε- carotene.

Chloropicaceae Lopes dos Santos and Eikrem fam. nov.

Diagnosis: With characters of order. Additional characters; cell wall thin and delicate.

*Chloropicon* Lopes dos Santos and Eikrem gen. nov.

Diagnosis: With characters of order. Coccoid cells measure 2–4 µm. One green chloroplast, often crescent shaped with starch grain. Thylakoids occur singly and in stacks of three. Central nucleus, mitochondrion located between nucleus and chloroplast. Vacuoles (1–2) present at cell periphery may contain particles. Surface of cell wall smooth.

Etymology: Named for its green color and small size.

Type species: *Chloropicon sieburthii*

*Chloropicon sieburthii* Lopes dos Santos and Eikrem sp. nov.

Diagnosis: With characters of the genus. Additional characters; combined nucleotide sequences of the nuclear 18S rRNA (AY425302), rRNA ITS (MF077490) and plastid 16S rRNA (AY702147) are species specific.

Holotype: Cells embedded in resin block deposited at the Natural History Museum, University of Oslo, accession number O-A-10001. Figure 2B–E show cells from the resin block. Authentic culture deposited in the Roscoff Culture Collection as RCC287.

Type locality: Strain RCC287 was isolated from water sampled in the Equatorial Pacific Ocean (0°, 179°49′ W) at 120 m depth.

Etymology: Named in honour of John McN. Sieburth, who published the first electron microscopy images of natural populations of marine picoeukaryotes.

*Chloropicon primus* Lopes dos Santos and Eikrem sp. nov.

Diagnosis: With characters of genus. Additional characters; combined nucleotide sequences of nuclear 18S rRNA (U40921), rRNA ITS (HE610139) and plastid 16S rRNA (AY702121, FN563080) are species specific.

Holotype: Cells embedded in resin block and thin-sections deposited at the Natural History Museum, University of Oslo, accession number O-A-10002. Figure 4B shows cell from the thin sections. Culture deposited in the Roscoff Culture Collection as RCC15.

Type locality: RCC15 was isolated in 1965 from a sample collected during the Trident cruise 26 in the Gulf Stream, North East Atlantic.

Etymology: The first species of the genus to be isolated into culture and have its 18S rRNA gene sequence published.

*Chloropicon roscoffensis* Lopes dos Santos and Eikrem sp. nov.

Diagnosis: With characters of genus. Additional characters; loroxanthin absent, combined nucleotide sequences of nuclear 18S rRNA (KF899840), rRNA ITS (MF077510) and plastid 16S rRNA (LN735295) are species specific.

Holotype: Cells embedded in resin block deposited at the Natural History Museum, University of Oslo accession number O-A-10003. Figure 4H shows a cell from the resin block. Culture deposited in the Roscoff Culture Collection as RCC1871.

Type locality: RCC1871 was isolated from the English Channel off Roscoff (48° 45′ N, 3° 57′ W).

Etymology: From the type locality.

*Chloropicon mariensis* Eikrem, Lopes dos Santos sp. nov.

Diagnosis: With characters of genus. Additional characters; combined nucleotide sequences of nuclear 18S rRNA (KF422632), rRNA ITS (MF077504) and plastid 16S rRNA (LN735516) are species specific.

Holotype: Cells embedded in resin block deposited at the Natural History Museum, University of Oslo, accession number O-A-10004. Figure 4F shows cell from the resin block. Culture deposited in the Roscoff Culture Collection as RCC998.

Type locality: RCC998 was isolated from water sampled at 100 m depth in the South Pacific Ocean (9° 04′ S, 136° 59′ W).

Etymology: Named in recognition of Dominique Marie who isolated the culture and his efforts in picoplankton research.

*Chloropicon laureae* Lopes dos Santos and Eikrem sp. nov.

Diagnosis: With characters of genus. Additional characters; combined nucleotide sequences of nuclear 18S rRNA (KF422631), rRNA ITS (MF077480) and plastid 16S rRNA (LN735470) are species specific.

Holotype: Cells embedded in resin block deposited at the Natural History Museum, University of Oslo, accession number O-A-10005. Figure 4D shows cell from the resin block. Culture deposited in the Roscoff Culture Collection as RCC856.

Type locality: RCC856 was isolated from water sampled at 10 m depth in the South Pacific Ocean off Marquesas Islands (8° 20′ S, 141° 15′ W).

Etymology: Named after Laure Guillou who first distinguished the prasinophyte clades, including clade VII.

*Chloropicon maureeniae* Lopes dos Santos and Eikrem sp. nov.

Diagnosis: With characters of genus. Additional characters; combined nucleotide sequences of nuclear 18S rRNA (KU843595), rRNA ITS (MF077515) and, plastid 16S rRNA (KU843568) are species specific.

Holotype: Cells embedded in resin block deposited at the Natural History Museum, University of Oslo, accession number O-A-10006. Figure 4J shows cell from the resin block. Culture deposited in the Roscoff Culture Collection as RCC3374.

Type locality: RCC3374 (CCMP2152) was isolated from the North Pacific Ocean off Hawaii (22° 45′ N, 158° 00′ W).

Etymology: Named in recognition of Maureen Keller who developed K medium that has facilitated the isolation of oceanic species into culture.

*Chloroparvula* Lopes dos Santos, Noël and Eikrem gen. nov.

Diagnosis: With characters of the family. Additional characters; cell wall thick and smooth or sometimes with fibrils, string like ornamentation.

Etymology: Named for its green color and small size.

Type species: *Chloroparvula pacifica*

*Chloroparvula pacifica* Lopes dos Santos, Noël and Eikrem sp. nov.

Diagnosis: With characters of genus with loroxanthin absent. Combined nucleotide sequences of nuclear 18S rRNA (KU843574), rRNA ITS (MF077486) and plastid 16S rRNA (KU843560) are species specific.

Holotype: Cells embedded in resin block deposited at the Natural History Museum, University of Oslo, accession number O-A-10007. Figure 3D shows cells from the resin block. Original culture deposited in NIES Microbial Culture Collection as NIES-3669; sub-culture deposited in Roscoff Culture Collection as RCC4656.

Type locality: NIES-3669 (RCC4656) was isolated from a surface water sample collected from the North Pacific Ocean off Japan (42°16′ N, 145°07′ E).

Etymology: Named for its abundance in the Pacific Ocean.

*Chloroparvula japonica* Lopes dos Santos, Noël and Eikrem sp. nov.

Diagnosis: With characters of genus. Additional characters; combined nucleotide sequences of nuclear 18S rRNA (KF422628), rRNA ITS (MF077482) and plastid 16S rRNA (LN735350) are species specific.

Holotype: Cells embedded in resin block deposited at the Natural History Museum, University of Oslo, accession number O-A-10008. Figure 3F shows cell from the resin block. Original culture deposited in NIES Microbial Culture Collection as NIES-2758; sub-culture deposited in Roscoff Culture Collection as RCC2339.

Type locality: NIES-2758 (RCC2339) was isolated in surface from the North Pacific Ocean off the coast of Japan (33° 46′ N, 129° 41′ E).

Etymology: Named for the origin of the authentic culture off the coast of Japan.

Picocystophyceae Eikrem and Lopes dos Santos classis nov.

Diagnosis: Green coccoid cells with chlorophylls *a* and *b*. Layered cell wall containing polyarabinose, mannose, galactose and glucose. Chloroplast surrounded by two membranes and containing starch grain.

Picocystales Eikrem and Lopes dos Santos order nov.

Diagnosis: With characters of class. Additional characters; accessory pigments are alloxanthin, diatoxanthin, monadoxanthin, chlorophyll *b*, neoxanthin, lutein and β,β- carotene.

Picocystaceae Eikrem and Lopes dos Santos fam. nov.

Diagnosis: With characters of order. Additional characters; coccoid cells contain green chloroplasts with starch grain.

*Picocysti*s R. A Lewin. Characters of type species *Picocystis salinarum*.

*Picocystis salinarum* R.A. Lewin emend. Eikrem and Lopes dos Santos.

Diagnosis: Cells measuring 2-3 µm with 1-2 chloroplasts, a mitochondrion and dictyosome. Combined nucleotide sequences of nuclear 18S rRNA (FR865649), rRNA ITS (HE610138, MF077484) and plastid 16S rRNA (AB491631) are species specific.Paratype: Cells embedded in resin block deposited at the Natural History Museum, University of Oslo, accession number O-A-10009. Supplement figure 2A shows cell from embedding. Original Culture CCMP1897 deposited in Roscoff Culture Collection as RCC3402.Type locality: Pacific ocean (37°47′N, 122°21′W).

### Availability of materials and data

All material including data, figures and tables are available from: 10.6084/m9.figshare.5027375.

## Electronic supplementary material

Supplementary Material

## References

[1] Tragin ML, dos Santos A, Christen R, Vaulot D (2016). Diversity and ecology of green microalgae in marine systems: an overview based on 18S rRNA gene sequences. Perspect. Phycol..

[2] Leliaert F (2012). Phylogeny and molecular evolution of the green algae. CRC. Crit. Rev. Plant Sci..

[3] Leliaert F, Verbruggen H, Zechman FW (2011). Into the deep: New discoveries at the base of the green plant phylogeny. BioEssays.

[4] Guillou L (2004). Diversity of picoplanktonic prasinophytes assessed by direct nuclear SSU rDNA sequencing of environmental samples and novel isolates retrieved from oceanic and coastal marine ecosystems. Protist.

[5] Lewin RA, Krienltz L, Oerickei RG, Takeda H, Hepperle D (2000). *Picocystis salinarum* gen. et sp. nov. (Chlorophyta) - a new picoplanktonic green alga. Phycologia.

[6] Roesler CS (2002). Distribution, production, and ecophysiology of *Picocystis* strain ML in Mono Lake, California. Limnol. Oceanogr..

[7] Krienitz L, Bock C, Kotut K, Luo W (2012). *Picocystis salinarum* (Chlorophyta) in saline lakes and hot springs of East Africa. Phycologia.

[8] Lopes dos Santos A, Gourvil P, Rodriguez-Hernandez F, Garrido JL, Vaulot D (2016). Photosynthetic pigments of oceanic Chlorophyta belonging to prasinophytes clade VII. J. Phycol..

[9] Lopes dos Santos A (2017). Diversity and oceanic distribution of prasinophytes clade VII, the dominant group of green algae in oceanic waters. ISME J..

[10] Marin B, Melkonian M (2010). Molecular phylogeny and classification of the Mamiellophyceae class. nov. (Chlorophyta) based on sequence comparisons of the nuclear- and plastid-encoded rRNA Operons. Protist.

[11] Marin B (2012). Nested in the Chlorellales or independent class? Phylogeny and classification of the Pedinophyceae (Viridiplantae) revealed by molecular phylogenetic analyses of complete nuclear and plastid-encoded rRNA operons. Protist.

[12] Lemieux C, Otis C, Turmel M (2014). Six newly sequenced chloroplast genomes from prasinophyte green algae provide insights into the relationships among prasinophyte lineages and the diversity of streamlined genome architecture in picoplanktonic species. BMC Genomics.

[13] Moon-van der Staay SY, De Wachter R, Vaulot D (2001). Oceanic 18S rDNA sequences from picoplankton reveal unsuspected eukaryotic diversity. Nature.

[14] Shi, X. L., Marie, D., Jardillier, L., Scanlan, D. J. & Vaulot, D. Groups without cultured representatives dominate eukaryotic picophytoplankton in the oligotrophic South East Pacific Ocean. *PLoS One* **4** (2009). 10.1371/journal.pone.0007657 PMC2764088 19893617

[15] Viprey M, Guillou L, Ferréol M, Vaulot D (2008). Wide genetic diversity of picoplanktonic green algae (Chloroplastida) in the Mediterranean Sea uncovered by a phylum-biased PCR approach. Environ. Microbiol..

[16] Wu W, Huang B, Liao Y, Sun P (2014). Picoeukaryotic diversity and distribution in the subtropical-tropical South China Sea. FEMS Microbiol. Ecol..

[17] Romari K, Vaulot D (2004). Composition and temporal variability of picoeukaryote communities at a coastal site of the English Channel from 18S rDNA sequences. Limnol. Oceanogr..

[18] Potter D, Lajeunesse TC, Saunders GW, Andersen RA (1997). Convergent evolution masks extensive biodiversity among marine coccoid picoplankton. Biodivers. Conserv..

[19] Leliaert F (2014). DNA-based species delimitation in algae DNA-based species delimitation in algae. Eur. J. Phycol..

[20] Mai JC, Coleman AW (1997). The internal transcribed spacer 2 exhibits a common secondary structure in green algae and flowering plants. J. Mol. Evol..

[21] Coleman AW (2000). The significance of a coincidence between evolutionary landmarks found in mating affinity and a Dna sequence. Protist.

[22] Coleman AW (2009). Is there a molecular key to the level of ‘biological species’ in eukaryotes? A DNA guide. Mol. Phylogenet. Evol..

[23] Joseph N, Krauskopf E, Vera MI, Michot B (1999). Ribosomal internal transcribed spacer 2 (ITS2) exhibits a common core of secondary structure in vertebrates and yeast. Nucleic Acids Res..

[24] Pöll, G. *et al*. rRNA maturation in yeast cells depleted of large ribosomal subunit proteins. *PLoS One* **4** (2009). 10.1371/journal.pone.0008249 PMC2788216 20011513

[25] Schillewaert S, Wacheul L, Lhomme F, Lafontaine DLJ (2012). The Evolutionarily Conserved Protein LAS1 Is Required for Pre-rRNA Processing at Both Ends of ITS2. Mol. Cell. Biol..

[26] Müller T, Philippi N, Dandekar T, Schultz J, Wolf M (2007). Distinguishing species. RNA.

[27] Guillard RRL, Hargraves PE (1993). *Stichochrysis immobilis* is a diatom, not a chrysophyte. Phycologia.

[28] Okaichi, T., Nishio, S. & Imatomi, Y. In *Toxic phytoplankton - occurrence*, *mode of action and toxins* (ed. Japanese Fisheries Society, T.)23–34 (1982).

[29] Zapata M, Rodriguez F, Garrido JL (2000). Separation of chlorophylls and carotenoids from marine phytoplankton: a new HPLC method using a reversed phase C-8 column and pyridine-containing mobile phases. Mar. Ecol. - Prog. Ser..

[30] Garrido JL, Rodríguez F, Zapata M (2009). Occurrence of loroxanthin, loroxanthin decenoate, and loroxanthin dodecenoate in *Tetraselmis* species (Prasinophyceae, Chlorophyta). J. Phycol..

[31] Schindelin J (2012). Fiji: an open-source platform for biological-image analysis. Nat. Methods.

[32] Marie, D., Simon, N., Guillou, L., Partensky, F. & Vaulot, D. DNA/RNA analysis of phytoplankton by flow cytometry. *Curr*. *Protoc*. *Cytom*. Chapter 11, Unit11.12 (2001). 10.1002/0471142956.cy1112s11 18770686

[33] Lepère C (2011). Whole Genome Amplification (WGA) of marine photosynthetic eukaryote populations. FEMS Microbiol. Ecol..

[34] White, T. J., Bruns, T., Lee, S. & Taylor, J. In *PCR protocols*: *a guide to methods and applications*. 315–322 (Academic Press, Orlando, Florida, 1990).

[35] West NJ (2001). Closely related *Prochlorococcus* genotypes show remarkably different depth distributions in two oceanic regions as revealed by *in situ* hybridization using 16S rRNA-targeted oligonucleotides. Microbiology.

[36] Fuller NJ (2006). Analysis of photosynthetic picoeukaryote diversity at open ocean sites in the Arabian Sea using a PCR biased towards marine algal plastids. Aquat. Microb. Ecol..

[37] Fuller NJ (2006). Molecular analysis of photosynthetic picoeukaryote community structure along an Arabian Sea transect. Limnol. Oceanogr..

[38] Keller A (2009). 5.8S–28S rRNA interaction and HMM-based ITS2 annotation. Gene.

[39] Ankenbrand MJ, Keller A, Wolf M, Schultz J, Föster F (2015). ITS2 database V: Twice as much. Mol. Biol. Evol..

[40] Zuker M (2003). Mfold web server for nucleic acid folding and hybridization prediction. Nucleic Acids Res..

[41] Coleman AW (2003). ITS2 is a double-edged tool for eukaryote evolutionary comparisons. Trends Genet..

[42] Coleman AW (2007). Pan-eukaryote ITS2 homologies revealed by RNA secondary structure. Nucleic Acids Res..

[43] Seibel PN, Müller T, Dandekar T, Schultz J, Wolf M (2006). 4SALE–a tool for synchronous RNA sequence and secondary structure alignment and editing. BMC Bioinformatics.

[44] Seibel P, Müller T, Dandekar T, Wolf M (2008). Synchronous visual analysis and editing of RNA sequence and secondary structure alignments using 4SALE. BMC Res. Notes.

[45] Caisová L, Marin B, Melkonian M (2013). A consensus secondary structure of ITS2 in the Chlorophyta identified by phylogenetic reconstruction. Protist.

[46] Kearse M (2012). Geneious Basic: An integrated and extendable desktop software platform for the organization and analysis of sequence data. Bioinformatics.

[47] Katoh K, Misawa K, Kuma K, Miyata T (2002). MAFFT: a novel method for rapid multiple sequence alignment based on fast Fourier transform. Nucleic Acids Res..

[48] Tamura K, Stecher G, Peterson D, Filipski A, Kumar S (2013). MEGA6: Molecular evolutionary genetics analysis version 6.0. Mol. Biol. Evol..

[49] Guindon S, Dufayard J-F, Lefort V, Anisimova M (2010). New algorithms and methods to estimate maximum- likelihoods phylogenies: Assessing the performance of PhyML 3.0. Syst. Biol..

[50] Ronquist F, Huelsenbeck JP (2003). MrBAYES 3: Bayesian phylogenetic inference under mixed models. Bioinformatics.

[51] Keeling PJ (2014). The Marine Microbial Eukaryote Transcriptome Sequencing Project (MMETSP): illuminating the functional diversity of eukaryotic life in the oceans through transcriptome sequencing. PLoS Biol.

[52] Schmieder R, Lim YW, Edwards R (2012). Identification and removal of ribosomal RNA sequences from metatranscriptomes. Bioinformatics.

[53] Pruesse E (2007). SILVA: a comprehensive online resource for quality checked and aligned ribosomal RNA sequence data compatible with ARB. Nucleic Acids Res..

[54] Grabherr MG (2011). Full-length transcriptome assembly from RNA-Seq data without a reference genome. Nat. Biotechnol..

[55] Trapnell C (2010). Transcript assembly and quantification by RNA-Seq reveals unannotated transcripts and isoform switching during cell differentiation. Nat. Biotechnol..

[56] Li B, Dewey CN (2011). RSEM: accurate transcript quantification from RNA-Seq data with or without a reference genome. BMC Bioinformatics.

[57] Parra G, Bradnam K, Korf I (2007). CEGMA: a pipeline to accurately annotate core genes in eukaryotic genomes. Bioinformatics.

[58] Goff SA (2011). The iPlant collaborative: cyberinfrastructure for plant biology. *Plant Genet*. Genomics.

[59] Tatusov RL (2003). The COG database: an updated version includes eukaryotes. BMC Bioinformatics.

[60] Castresana J (2000). Selection of conserved blocks from multiple alignments for their use in phylogenetic analysis. Mol. Biol. Evol..

[61] Darriba D, Taboada GL, Doallo R, Posada D (2011). ProtTest 3: fast selection of best-fit models of protein evolution. Bioinformatics.

[62] Huelsenbeck JP, Ronquist F (2001). MRBAYES: Bayesian inference of phylogenetic trees. Bioinformatics.

[63] Derelle E (2006). Genome analysis of the smallest free-living eukaryote *Ostreococcus tauri* unveils many unique features. Proc. Natl. Acad. Sci. USA.

[64] Worden AZ (2009). Green evolution and dynamic adaptations revealed by genomes of the marine picoeukaryotes *Micromonas*. Science.

[65] Moreau H (2012). Gene functionalities and genome structure in *Bathycoccus prasinos* reflect cellular specializations at the base of the green lineage. Genome Biol..

[66] Subirana L (2013). Morphology, genome plasticity, and phylogeny in the genus *Ostreococcus* reveal a cryptic species, *O*. *mediterraneus* sp. nov. (Mamiellales, Mamiellophyceae). Protist.

[67] Wang B, Zarka A, Trebst A, Boussiba S (2003). Astaxanthin accumulation in *Haematococcus pluvialis* (Chlorophyceae) as an active photoprotective process under high irradiance. J. Phycol..

[68] Jeffrey, S. W., Wright, S. W. & Zapata, M. In *Phytoplankton Pigments*: *Characterization*, *Chemotaxonomy and Applications in Oceanography* (ed. Roy S, Egeland ES, Johnsen G, L. C.) 3–77 (Cambridge University Press, Cambridge., 2011).

[69] Rodríguez F (2005). Ecotype diversity in the marine picoeukaryote *Ostreococcus* (Chlorophyta, Prasinophyceae). Environ. Microbiol..

[70] Latasa M, Scharek R, Le Gall F, Guillou L (2004). Pigment suites and taxonomic groups in Prasinophyceae. J. Phycol..

[71] Takaichi S (2011). Carotenoids in Algae: Distributions, biosyntheses and functions of carotenoids in algae. Mar. Drugs.

[72] Crespo C, Rodríguez H, Segade P, Iglesias R, García-Estévez JM (2009). *Coccomyxa* sp. (Chlorophyta: Chlorococcales), a new pathogen in mussels (*Mytilus galloprovincialis*) of Vigo estuary (Galicia, NW Spain). J. Invertebr. Pathol..

[73] Jahns P, Latowski D, Strzalka K (2009). Mechanism and regulation of the violaxanthin cycle: The role of antenna proteins and membrane lipids. Biochim. Biophys. Acta - Bioenerg..

[74] Lemieux C, Otis C, Turmel M (2014). Chloroplast phylogenomic analysis resolves deep-level relationships within the green algal class Trebouxiophyceae. BMC Evol. Biol..

[75] Leliaert F (2016). Chloroplast phylogenomic analyses reveal the deepest-branching lineage of the Chlorophyta, Palmophyllophyceae class. nov. Sci. Rep..

[76] Marie D, Le Gall F, Edern R, Gourvil P, Vaulot D (2017). Improvement of phytoplankton culture isolation using single cell sorting by flow cytometry. J. Phycol..

[77] Simon, N. *et al*. Revision of the genus *Micromonas* (Manton et Parke) (Chlorophyta, Mamiellophyceae), of the type species *M*. *pusilla* (Butcher) Manton & Parke and of the species *M*. *commoda* (van Baren, Bacry and Worden) and description of two new species based on the genetic and phenotypic characterization of cultured isolates. *Protist* in press (2017). 10.1016/j.protis.2017.09.002 29028580

[78] Schultz J, Maisel S, Gerlach D, Müller T, Wolf M (2005). A common core of secondary structure of the internal transcribed spacer 2 (ITS2) throughout the Eukaryota. RNA.

[79] Caisová L, Marin B, Melkonian M (2011). A close-up view on ITS2 evolution and speciation - a case study in the Ulvophyceae (Chlorophyta, Viridiplantae). BMC Evol. Biol..

[80] Kaczmarska I, Mather L, Aluddington I, Muise F, Ehrman JM (2014). Cryptic diversity in a cosmopolitan diatom known as *Asterionellopsis glacialis* (Fragilariaceae): Implications for ecology, biogeography, and taxonomy. Am. J. Bot..

[81] Nakayama T (1998). The basal position of scaly green flagellates among the green algae (Chlorophyta) is revealed by analyses of nuclear-encoded SSU rRNA sequences. Protist.

[82] Massjuk NP (2006). Chlorodendrophyceae class. nov. (Chlorophyta, Viridiplantae) in the Ukrainian flora: I. The volume, phylogenetic relations and taxonomical status. Ukr. Bot. J..

[83] Guillard RRL, Keller MD, O’Kelly CJ, Floyd GL (1991). *Pycnococcus provasolii* gen. et sp. nov., a coccoid prasinoxanthin containing phytoplankter from the western North Atlantic and Gulf of Mexico. J. Phycol..

[84] Jouenne F, Eikrem W, Le Gall F, Johnsen G, Vaulot D (2011). *Prasinoderma singularis* sp. nov., a solitary coccoid prasinophyte from the South East Pacific Ocean. Protist.

[85] Fawley MW, Qin M, Yun Y (1999). The relationship between *Pseudoscourfieldia marina* and *Pycnococcus provasolii* (Prasinophyceae, Chlorophyta): Evidence from 18S rDNA sequence data. J. Phycol..

[86] Henley WJ (2004). Phylogenetic analysis of the ‘*Nannochloris*-like’ algae and diagnoses of *Picochlorum oklahomensis* gen. et sp nov (Trebouxiophyceae, Chlorophyta). Phycologia.

[87] Balzano S (2017). Morphological and genetic diversity of Beaufort Sea diatoms with high contributions from the *Chaetoceros neogracilis* species complex. J. Phycol..

[88] Percopo I (2016). *Pseudo-nitzschia arctica* sp. nov., a new cold-water cryptic *Pseudo-nitzschia* species within the *P*. *pseudodelicatissima* complex. J. Phycol..

[89] Rodríguez-Martínez R, Rocap G, Logares R, Romac S, Massana R (2012). Low evolutionary diversification in a widespread and abundant uncultured protist (MAST-4). Mol. Biol. Evol..

