# Author Correction: Chloropicophyceae, a new class of picophytoplanktonic prasinophytes

**DOI:** 10.1038/s41598-020-63703-3

**Published:** 2020-04-17

**Authors:** Adriana Lopes dos Santos, Thibaut Pollina, Priscillia Gourvil, Erwan Corre, Dominique Marie, José Luis Garrido, Francisco Rodríguez, Mary-Hélène Noël, Daniel Vaulot, Wenche Eikrem

**Affiliations:** 1 Sorbonne Universités, UPMC Université Paris 06, CNRS, UMR7144, Station Biologique de Roscoff, Roscoff, France; 2 Norwegian Institute for Water Research, Gaustadalléen 21, 0349 Oslo, Norway; 3 Instituto de Investigaciones Marinas (CSIC), Av. Eduardo Cabello, 6, 36208 Vigo, Spain; 4 Instituto Español de Oceanografia (IEO), Centro Oceanográfico de Vigo, Subida a Radio Faro, 36390 Vigo, Spain; 5 Centro de Genómica y Bioinformática, Facultad de Ciencias, Universidad Mayor. Camino La Pirámide, 5750 Huechuraba, Santiago Chile; 6 National Institute for Environmental Studies, Tsukuba, Japan; 7 Natural History Museum, University of Oslo, PO Box 1069, Blindern, 0316 Oslo Norway; 8 Department of Biosciences, University of Oslo, PO Box 1066, Blindern, 0316 Oslo Norway

Correction to: *Scientific Reports* 10.1038/s41598-017-12412-5, published online 25 October 2017

The original version of this Article omitted an affiliation for Thibaut Pollina and Wenche Eikrem. The correct affiliations are listed below:

Thibaut Pollina:

Sorbonne Universités, UPMC Université Paris 06, CNRS, UMR7144, Station Biologique de Roscoff, Roscoff, France

Norwegian Institute for Water Research, Gaustadalléen 21, 0349, Oslo, Norway

Department of Biosciences, University of Oslo, PO Box 1066, Blindern, 0316, Oslo, Norway

Wenche Eikrem:

Norwegian Institute for Water Research, Gaustadalléen 21, 0349, Oslo, Norway

Natural History Museum, University of Oslo, PO Box 1069, Blindern, 0316, Oslo, Norway

Department of Biosciences, University of Oslo, PO Box 1066, Blindern, 0316, Oslo, Norway

This has now been corrected in the HTML and PDF versions of this Article, and in the accompanying Supplemental Material.

