# Supplementary material for: Chloropicophyceae, a new class of picophytoplanktonic prasinophytes
Source: Sci Rep. 2017 Oct 25;7:14019. doi: 10.1038/s41598-017-12412-5 (PMC5656628; doi:10.1038/s41598-017-12412-5)
Supplement: Supplementary file 1 — Supplementary Material [file 41598_2017_12412_MOESM1_ESM.pdf]

Chloropicophyceae, a new class of picophytoplanktonic prasinophytes.

Supplementary Material

Adriana Lopes dos Santos [1,5] , Thibaut Pollina [1,2,8], Priscillia Gourvil [1], Erwan Corre [1], Dominique Marie [1], José Luis Garrido [3], Francisco Rodríguez [4], Mary-Hélène Noël [6], Daniel Vaulot [1] and Wenche Eikrem [2,7,8]

1 Sorbonne Universités, UPMC Université Paris 06, CNRS, UMR7144, Station Biologique de Roscoff, Roscoff, France.

2 Norwegian Institute for Water Research, Gaustadalléen 21, 0349, Oslo, Norway.

3 Instituto de Investigaciones Marinas (CSIC). Av. Eduardo Cabello, 6. 36208, Vigo, Spain.

4 Instituto Español de Oceanografia (IEO), Centro Oceanográfico de Vigo, Subida a Radio Faro, 36390, Vigo, Spain.

5 Centro de Genómica y Bioinformática, Facultad de Ciencias, Universidad Mayor. Camino La Pirámide 5750, Huechuraba, Santiago, Chile.

6 National Institute for Environmental Studies, Tsukuba, Japan.

7 Natural History Museum, University of Oslo, PO Box 1069, Blindern, 0316 Oslo, Norway.

8 Department of Biosciences, University of Oslo, PO Box 1066, Blindern, 0316 Oslo, Norway

[1]Corresponding author: lopesas.ufrj@gmail.com

Key words: Chlorophyta, prasinophytes, clade VII, Chloropicophyceae, Picocystophyceae

Supplementary table legends

Supplementary Table 1: List of primers used in this study

Supplementary Table 2: List of transcriptomes from the Marine Microbiology Initiative (MMETSP) used in this study.

Supplementary Table 3: List of KOGs used for the multigene alignment.

Supplementary Table 4: Average of uncorrected *p*-distance based on the concatenated nuclear and plastid SSU rRNA sequences and ITS2 (in parenthesis).

Supplementary Table 5: List of changes CBCs, hCBCs and non-CBC (e.g. N – N ↔ N x N) detected in all the helices. Each change is given a ID number (second column) and the position of each change is provided following the alignment positions (third column).

Supplementary Table 1

						Initial Denaturation			Denaturation		Annealing		Extension		Elongation	
**Gene**	**Primer forward**	**Sequence**	**Primer reverse**	**Sequence**	**Reference**	**N cycles**	**Time**	**Temperature**	**Time**	**Temperature**	**Time**	**Temperature**	**Time**	**Temperature**	**Time**	**Temperature**
18S rRNA	Euk63F	5' - ACGCTTGTCTCAAAGATTA - 3'	Euk1818R	5' - ACGGAAACCTTGTTACGA - 3'	Lepère *et* al. 2011	35	30 s	98°C	10 s	98°C	30 s	55°C	60 s	72°C	10 min	72°C
ITS1 - 5.8S - ITS2	ITS5	5' - GAAAGTAAAAGTCGTAACAAGG - 3'	ITS4	5' - TCCTCCGCTTATTGATATGC - 3'	White *et* al. 1990	35	30 s	98°C	10 s	98°C	30s	52°C	1 min	72°C	10 min	72°C
plastid 16S rRNA	PLA491F	5' - GAGGAATAAGCATCGGCTAA - 3'	OXY1313R	5' - CTTCAYGYAGGCGAGTTGCAGC - 3'	Fuller *et* al. 2006 and West *et* al. 2001	35	30 s	98°C	10 s	98°C	30 s	60°C	30 s	72°C	10 min	72°C

Supp Table 2

Transcriptome	Class	Family	Genus	Species	Clade	Strain	RCC
MMETSP0491	Chlorodendrophyceae	Chlorodendraceae	Tetraselmis	chuii		PLY429	
MMETSP0817	Chlorodendrophyceae	Chlorodendraceae	Tetraselmis	striata		LANL1001	
MMETSP0063	Chlorophyceae	Chlamydomonadaceae	Chlamydomonas	euryale		CCMP219	
MMETSP0052	Chlorophyceae	Chlamydomonadaceae	Polytomella	parva		SAG 63-3	
MMETSP1126	Chlorophyceae	Dunaliellaceae	Dunaliella	tertiolecta		CCMP1320	
MMETSP1310	Chloropicophyceae		Chloroparvula	japonica	B	NIES-2758	2339
MMETSP1311	Chloropicophyceae		Chloropicon	laureae	A5	RCC856	856
MMETSP1453	Chloropicophyceae		Chloropicon	laureae	A5	RCC701	701
MMETSP1309	Chloropicophyceae		Chloropicon	mariensis	A1	RCC998	998
MMETSP1469	Chloropicophyceae		Chloropicon	primus	A2	CCMP1205	15
MMETSP1085	Chloropicophyceae		Chloropicon	roscoffensis	A4	CCMP1998	
MMETSP1312	Chloropicophyceae		Chloropicon	roscoffensis	A4	NIES-2755	2335
MMETSP1456	Chloropicophyceae		Chloropicon	roscoffensis	A4	RCC1871	1871
MMETSP1446	Chloropicophyceae		Chloropicon	sp.	A	CCMP2111	3368
MMETSP1399	Mamiellophyceae	Bathycoccaceae	Bathycoccus	prasinos		CCMP1898	
MMETSP0939	Mamiellophyceae	Bathycoccaceae	Ostreococcus	lucimarinus	A	BCC118000	
MMETSP0930	Mamiellophyceae	Bathycoccaceae	Ostreococcus	mediterraneus	D	RCC1621	1621
MMETSP0803	Mamiellophyceae	Dolichomastigaceae	Crustomastix	stigmata		CCMP3273	
MMETSP0033	Mamiellophyceae	Dolichomastigaceae	Dolichomastix	tenuilepis		CCMP3274	
MMETSP1326	Mamiellophyceae	Mamiellaceae	Genus nov.	species nov.		RCC2288	2288
MMETSP1106	Mamiellophyceae	Mamiellaceae	Mantoniella	antarctica		SL-175	
MMETSP1468	Mamiellophyceae	Mamiellaceae	Mantoniella	sp.		CCMP1436	
MMETSP1080	Mamiellophyceae	Mamiellaceae	Micromonas	bravo	B	CCMP1646	806
MMETSP1327	Mamiellophyceae	Mamiellaceae	Micromonas	polaris		RCC2306	2306
MMETSP0034	Nephroselmidophyceae		Nephroselmis	pyriformis		CCMP717	
MMETSP0941	Palmophyllophyceae		Prasinococcus	capsulatus		CCMP1194	
MMETSP0806	Palmophyllophyceae		Prasinoderma	coloniale		CCMP1413	
MMETSP1315	Palmophyllophyceae		Prasinoderma	singularis		RCC927	927
MMETSP0807	Picocystophyceae		Picocystis	salinarum	C	CCMP1897	
MMETSP0804	Prasinophyceae	Chlorodendraceae	Tetraselmis	astigmatica		CCMP880	
MMETSP0419	Prasinophyceae	Chlorodendraceae	Tetraselmis	sp.		GSL018	
MMETSP1401	Prasinophyceae	Mamiellaceae	Micromonas	bravo	B	CCAC1681	
MMETSP1082	Prasinophyceae	Mamiellaceae	Micromonas	commoda	A	NEPCC29	804
MMETSP1387	Prasinophyceae	Mamiellaceae	Micromonas	commoda	A	RCC472	472
MMETSP1390	Prasinophyceae	Mamiellaceae	Micromonas	polaris		CCMP2099	
MMETSP0929	Prasinophyceae	Mamiellaceae	Ostreococcus	mediterraneus	D	RCC2572	2572
MMETSP1438	Prasinophyceae	Pterospermataceae	Pterosperma	sp.		CCMP1384	
MMETSP1316	Prasinophyceae	Pycnococcaceae	Pycnococcus	provasolii		RCC2336	2336
MMETSP1459	Prasinophyceae	Pycnococcaceae	Pycnococcus	provasolii		RCC931	931
MMETSP1081	Prasinophyceae	Pyramimonadaceae	Pyramimonas	amylifera		CCMP720	
MMETSP0058	Prasinophyceae	Pyramimonadaceae	Pyramimonas	parkeae		CCMP726	
MMETSP1169	Prasinophyceae		Pyramimonas	obovata		CCMP722	
MMETSP1445	Prasinophyceae		Pyramimonas	sp.		CCMP2087	
MMETSP1161	Trebouxiophyceae		Picochlorum	oklahomensis		CCMP2329	
MMETSP1473	Trebouxiophyceae		Stichococcus	sp.		RCC1054	1054

Supplementary Table 3

KOG	Function
KOG0003	Ubiquitin/60s ribosomal protein L40 fusion
KOG0019	Molecular chaperone (HSP90 family)
KOG0073	GTP-binding ADP-ribosylation factor-like protein ARL2
KOG0077	Vesicle coat complex COPII, GTPase subunit SAR1
KOG0084	GTPase Rab1/YPT1, small G protein superfamily, and related GTP-binding proteins
KOG0092	GTPase Rab5/YPT51 and related small G protein superfamily GTPases
KOG0102	Molecular chaperones mortalin/PBP74/GRP75, HSP70 superfamily
KOG0173	20S proteasome, regulatory subunit beta type PSMB7/PSMB10/PUP1
KOG0174	20S proteasome, regulatory subunit beta type PSMB6/PSMB9/PRE3
KOG0176	20S proteasome, regulatory subunit alpha type PSMA5/PUP2
KOG0177	20S proteasome, regulatory subunit beta type PSMB2/PRE1
KOG0179	20S proteasome, regulatory subunit beta type PSMB1/PRE7
KOG0180	20S proteasome, regulatory subunit beta type PSMB3/PUP3
KOG0181	20S proteasome, regulatory subunit alpha type PSMA2/PRE8
KOG0182	20S proteasome, regulatory subunit alpha type PSMA6/SCL1
KOG0183	20S proteasome, regulatory subunit alpha type PSMA7/PRE6
KOG0185	20S proteasome, regulatory subunit beta type PSMB4/PRE4
KOG0211	Protein phosphatase 2A regulatory subunit A and related proteins
KOG0233	Vacuolar H+-ATPase V0 sector, subunit c''
KOG0258	Alanine aminotransferase
KOG0279	G protein beta subunit-like protein
KOG0327	Translation initiation factor 4F, helicase subunit (eIF-4A) and related helicases
KOG0328	Predicted ATP-dependent RNA helicase FAL1, involved in rRNA maturation, DEAD-box superfamily
KOG0329	ATP-dependent RNA helicase
KOG0371	Serine/threonine protein phosphatase 2A, catalytic subunit
KOG0372	Serine/threonine specific protein phosphatase involved in glycogen accumulation, PP2A-related
KOG0373	Serine/threonine specific protein phosphatase involved in cell cycle control, PP2A-related
KOG0397	60S ribosomal protein L11
KOG0400	40S ribosomal protein S13
KOG0402	60S ribosomal protein L37
KOG0407	40S ribosomal protein S14
KOG0419	Ubiquitin-protein ligase
KOG0420	Ubiquitin-protein ligase
KOG0460	Mitochondrial translation elongation factor Tu
KOG0466	Translation initiation factor 2, gamma subunit (eIF-2gamma GTPase)
KOG0534	NADH-cytochrome b-5 reductase
KOG0544	FKBP-type peptidyl-prolyl cis-trans isomerase
KOG0556	Aspartyl-tRNA synthetase
KOG0659	Cdk activating kinase (CAK)/RNA polymerase II transcription initiation/nucleotide excision repair factor TFIIH/TFIIK, kinase subun
KOG0679	Actin-related protein - Arp4p/Act3p
KOG0734	AAA+-type ATPase containing the peptidase M41 domain
KOG0829	60S ribosomal protein L18A
KOG0857	60s ribosomal protein L10
KOG0878	60S ribosomal protein L32
KOG0880	Peptidyl-prolyl cis-trans isomerase
KOG0898	40S ribosomal protein S15
KOG0934	Clathrin adaptor complex, small subunit
KOG0938	Adaptor complexes medium subunit family
KOG0960	Mitochondrial processing peptidase, beta subunit, and related enzymes (insulinase superfamily)
KOG1088	Uncharacterized conserved protein
KOG1299	Vacuolar sorting protein VPS45/Stt10 (Sec1 family)
KOG1342	Histone deacetylase complex, catalytic component RPD3
KOG1350	F0F1-type ATP synthase, beta subunit
KOG1351	Vacuolar H+-ATPase V1 sector, subunit B
KOG1355	Adenylosuccinate synthase
KOG1373	Transport protein Sec61, alpha subunit
KOG1430	C-3 sterol dehydrogenase/3-beta-hydroxysteroid dehydrogenase and related dehydrogenases
KOG1439	RAB proteins geranylgeranyltransferase component A (RAB escort protein)
KOG1448	Ribose-phosphate pyrophosphokinase
KOG1463	26S proteasome regulatory complex, subunit RPN6/PSMD11
KOG1494	NAD-dependent malate dehydrogenase
KOG1498	26S proteasome regulatory complex, subunit RPN5/PSMD12

KOG1531	F0F1-type ATP synthase, gamma subunit
KOG1533	Predicted GTPase
KOG1555	26S proteasome regulatory complex, subunit RPN11
KOG1626	Inorganic pyrophosphatase/Nucleosome remodeling factor, subunit NURF38
KOG1641	Mitochondrial chaperonin
KOG1644	U2-associated snRNP A' protein
KOG1646	40S ribosomal protein S6
KOG1654	Microtubule-associated anchor protein involved in autophagy and membrane trafficking
KOG1678	60s ribosomal protein L15
KOG1712	Adenine phosphoribosyl transferases
KOG1722	60s ribosomal protein L24
KOG1723	60s ribosomal protein L30 isolog
KOG1733	Mitochondrial import inner membrane translocase, subunit TIM13
KOG1742	60s ribosomal protein L15/L27
KOG1753	40S ribosomal protein S16
KOG1754	40S ribosomal protein S15/S22
KOG1758	Mitochondrial F1F0-ATP synthase, subunit delta/ATP16
KOG1760	Molecular chaperone Prefoldin, subunit 4
KOG1775	U6 snRNA-associated Sm-like protein
KOG1779	40s ribosomal protein S27
KOG1780	Small Nuclear ribonucleoprotein G
KOG2309	60s ribosomal protein L2/L8
KOG2467	Glycine/serine hydroxymethyltransferase
KOG2509	Seryl-tRNA synthetase
KOG2670	Enolase
KOG2738	Putative methionine aminopeptidase
KOG2775	Metallopeptidase
KOG2916	Translation initiation factor 2, alpha subunit (eIF-2alpha)
KOG2930	SCF ubiquitin ligase, Rbx1 component
KOG3049	Succinate dehydrogenase, Fe-S protein subunit
KOG3052	Cytochrome c1
KOG3079	Uridylate kinase/adenylate kinase
KOG3090	Prohibitin-like protein
KOG3106	ER lumen protein retaining receptor
KOG3157	Proline synthetase co-transcribed protein
KOG3189	Phosphomannomutase
KOG3204	60S ribosomal protein L13a
KOG3222	Inosine triphosphate pyrophosphatase
KOG3271	Translation initiation factor 5A (eIF-5A)
KOG3275	Zinc-binding protein of the histidine triad (HIT) family
KOG3295	60S Ribosomal protein L13
KOG3301	Ribosomal protein S4
KOG3311	Ribosomal protein S18
KOG3320	40S ribosomal protein S7
KOG3343	Vesicle coat complex COPI, zeta subunit
KOG3361	Iron binding protein involved in Fe-S cluster formation
KOG3387	60S ribosomal protein 15.5kD/SNU13, NHP2/L7A family (includes ribonuclease P subunit p38), involved in splicing
KOG3405	RNA polymerase subunit K
KOG3406	40S ribosomal protein S12
KOG3411	40S ribosomal protein S19
KOG3418	60S ribosomal protein L27
KOG3436	60S ribosomal protein L35
KOG3453	Cytochrome c
KOG3459	Small nuclear ribonucleoprotein (snRNP) Sm core protein
KOG3464	60S ribosomal protein L44
KOG3475	60S ribosomal protein L37
KOG3479	Mitochondrial import inner membrane translocase, subunit TIM9
KOG3482	Small nuclear ribonucleoprotein (snRNP) SMF
KOG3489	Mitochondrial import inner membrane translocase, subunit TIM8
KOG3493	Ubiquitin-like protein
KOG3498	Preprotein translocase, gamma subunit
KOG3499	60S ribosomal protein L38
KOG3502	40S ribosomal protein S28
KOG3503	H/ACA snoRNP complex, subunit NOP10
KOG4655	U3 small nucleolar ribonucleoprotein (snoRNP) component

Supplementary Table 4

	A1	A2	A3	A4	A5	A6	A7	B1	B2	B3	*Picocystis salinarum*	NIES-2758	RCC3368	RCC3376	RCC996	Chlorodendrophyceae	Chlorophyceae	Pedinophyceae	Trebouxiophyceae	Ulvophyceae
**A1**	0.001 (0.001)																			
**A2**	0.013 (0.129)	0.002 (0.005)																		
**A3**	0.014 (0.128)	0.010 (0.112)	0.002 (0.002)																	
**A4**	0.016 (0.116)	0.011 (0.077)	0.010 (0.103)	0.001 (0)																
**A5**	0.013 (0.116)	0.015 (0.145)	0.012 (0.136)	0.014 (0.143)	0.001 (0)															
**A6**	0.032 (0.224)	0.032 (0.240)	0.029 (0.204)	0.032 (0.211)	0.033 (0.204)	0 (0)														
**A7**	0.039 (0.231)	0.043 (0.252)	0.042 (0.264)	0.042 (0.238)	0.041 (0.238)	0.035 (0.259)	0 (0)													
**B1**	0.130 (0.401)	0.131 (0.417)	0.129 (0.407)	0.130 (0.408)	0.129 (0.415)	0.117 (0.456)	0.122 (0.449)	0 (0)												
**B2**	0.128 (0.385)	0.129 (0.411)	0.134 (0.348)	0.133 (0.383)	0.135 (0.381)	0.119 (0.422)	0.129 (0.390)	0.074 (0.331)	0.004 (0.059)											
**B3**	0.120 *	0.121 *	0.125 *	0.123 *	0.123 *	0.110 *	0.120 *	0.077 *	0.022 *	0 *										
Picocystis salinarum	0.116 *	0.115 *	0.113 *	0.113 *	0.116 *	0.104 *	0.111 *	0.120 *	0.115 *	0.117 *	0 *									
**NIES-2758**	0.119 (0.401)	0.119 (0.376)	0.119 (0.381)	0.119 (0.361)	0.121 (0.388)	0.111 (0.442)	0.115 (0.442)	0.080 (0.313)	0.049 (0.186)	0.037 *	0.103*	0 (0)								
**RCC3368**	0.037 (0.170)	0.042 (0.177)	0.043 (0.157)	0.039 (0.163)	0.041 (0.190)	0.034 (0.245)	0.039 (0.224)	0.122 (0.422)	0.130 (0.392)	0.122*	0.110*	0.116 (0.429)	0 (0)							
**RCC3376**	0.030 (0.204)	0.035 (0.179)	0.037 (0.185)	0.035 (0.170)	0.034 (0.170)	0.034 (0.252)	0.039 (0.218)	0.118 (0.429)	0.120 (0.408)	0.112 *	0.110*	0.111 (0.429)	0.035 (0.197)	0 (0)						
**RCC996**	0.012 (0.122)	0.014 (0.124)	0.016 (0.171)	0.018 (0.129)	0.018 (0.156)	0.028 (0.259)	0.039 (0.252)	0.125 (0.408)	0.129 (0.406)	0.120 *	0.114*	0.116 (0.408)	0.034 (0.184)	0.034 (0.197)	0 (0)					
**Chlorodendrophyceae**	0.125*	0.127*	0.125*	0.126*	0.128*	0.120*	0.127*	0.137*	0.141*	0.136*	0.106	0.125*	0.124*	0.124*	0.124*	0.028*				
**Chlorophyceae**	0.150*	0.147*	0.148*	0.146*	0.150*	0.142*	0.147*	0.151*	0.153*	0.151*	0.131*	0.141*	0.150*	0.143*	0.146*	0.112*	0.078*			
**Pedinophyceae**	0.141*	0.140*	0.143*	0.143*	0.141*	0.131*	0.142*	0.135*	0.131	0.129*	0.114*	0.122*	0.141*	0.132*	0.139*	0.097*	0.119*	0.027*		
**Trebouxiophyceae**	0.141*	0.140*	0.138*	0.140*	0.140*	0.132*	0.143*	0.144*	0.143	0.142*	0.113*	0.138*	0.139*	0.132*	0.141*	0.097*	0.121*	0.107*	0.066*	
**Ulvophyceae**	0.142*	0.142*	0.141*	0.140*	0.142*	0.136*	0.146*	0.147*	0.148	0.152*	0.126*	0.144*	0.143*	0.140*	0.145*	0.105*	0.123*	0.116*	0.115*	0.034*

* ITS2 sequence not available

Supplementary Figure 5

Helix	Nucleotide pair id	Alignment positions of nucleotide pair	Remarks	*C. mariensis* (A1)	*C. primus* (A2)	*C. sieburthii* (A3)	*C. roscoffensis* (A4)	*C. laureae* (A5)	RCC4434 (A6)	*C. maureeniae* (A7)	RCC996 (A)	RCC3368 (A)	RCC3376 (A)	RCC2337 (B2)	RCC999 (B2)	RCC696 (B2)	*C. japonica* (NIES-2758)	*C. pacifica* (B1)
B9	1	16 - 384		G - U	A x C	A - U	A - U	A - U	A x C	G - U	A - U	G - C	A - U	A x C	A x C	A x C	A x C	A - U
	2	17 - 382		C x U	C x U	C x U	C x U	C x C	C x U	C x U	C x C	C x C	C x C	A - U	A - U	A - U	A x C	G - U
	3	20 - 379		G - C	A - U	A - U	A - U	G - C	A - U	G - C	G - C	G - C	G - C	G x A	G x A	G x A	G - U	G - C
	4	21 - 378		U - A	U - A	U - A	U - A	U - A	U - A	U - A	U - A	U - A	U - A	A - U	A - U	A - U	A - U	A x A
	5	22 - 376		A - U	A - U	A - U	A - U	A - U	A - U	A - U	A - U	U x U	U x U	A - U	A - U	A - U	A - U	U x U
I	6	27 - 97		G - U	G - U	G - U	G - U	G - U	G - U	G - U	G - U	G - U	G - U	G - U	G - U	G - U	G - C	G - U
	7	29 - 95		A x C	G - C	A x C	G - C	G - U	G - C	G - C	G - C	G - C	G - C	A - U	A - U	A - U	C x C	A x -
	8	31 - 93		A - U	A - U	A - U	A - U	A - U	A x -	A - U	A - U	A - U	A - U	U - A	U - A	U - A	U - A	A - U
	9	34 - 91		U - A	C - G	C - G	C - G	U - A	U - A	C - G	C x U	A - U	U - A	C - G	C - G	C - G	C - G	C - G
	10	38 - 90		U - G	U - A	U - A	U - G	U - A	U - A	A - U	C - G	A - U	C x C	U - A	U - A	U - A	C x A	U x C
	11	41 - 88		C - G	U - G	U - G	U - G	U - G	U - G	U - G	C - G	U - G	U - G	C - G	C - G	C - G	C - G	C x A
	12	42 - 83		C - G	C - G	C - G	C - G	C - G	C - G	C - G	U - G	C - G	C - G	C - G	C - G	C - G	C - G	C - G
	13	44 - 78		U - G	A - U	C - G	A - U	U - A	C - G	C - G	U - A	A - U	C - G	C - G	C - G	C - G	C - G	C - G
II	14	103 - 153		A - U	A - U	A - U	A - U	A - U	A - U	G - C	A - U	A - U	A - U	G - C	G - C	G - C	G - C	G - C
	15	105 - 151		C - G	C - G	C - G	C - G	C - G	C - G	U - A	C - G	C - G	C x A	C - G	C - G	C - G	C - G	C - G
	16	106 - 150	CBC A and B	U - G	U - G	U - G	U - G	U - G	U - G	C - G	U - G	U - G	U - G	G - C	G - C	G - C	G - C	G - C
	17	112 - 146		C - G	C - G	C - G	C - G	C - G	U - G	C - G	C - G	C - G	C - G	C - G	C - G	C - G	C - G	C x C
	18	113 - 145		A - U	A - U	A - U	A - U	A - U	U - G	A - U	A - U	A - U	A - U	G - C	G - C	G - C	G - C	G x G
	19	115 - 144		G - C	G - C	G - C	G - C	G - C	U - A	G - C	G - C	G - C	U - G	G - C	G - C	G - C	G - C	C - G
	20	117 - 142		A - U	G - U	C - G	G - U	U - A	C - G	G - U	G - U	G - U	G - U	A - U	A - U	A - U	G - C	G - C
	21	120 - 141	CBC B2	n.c	n.c	n.c	n.c	n.c	n.c	n.c	n.c	n.c	n.c	C - G	C - G	A - U	G - C	A - U
	22	123 - 136	CBC B2	n.c	n.c	n.c	n.c	n.c	n.c	n.c	n.c	n.c	n.c	U - A	U - A	U - A	C - G	C - G
	23	124 - 135	CBC B2	n.c	n.c	n.c	n.c	n.c	n.c	n.c	n.c	n.c	n.c	U - A	U - A	U - A	C - G	C - G
	24	126 - 133	CBC B2	n.c	n.c	n.c	n.c	n.c	n.c	n.c	n.c	n.c	n.c	C - G	C - G	C - G	C - G	G - C
	25	127 - 132		n.c	n.c	n.c	n.c	n.c	n.c	n.c	n.c	n.c	n.c	G - C	G - C	G - C	G x G	A - U
III	26	176 - 334		G - C	G - C	G - C	G - C	G - C	G - C	G - C	G - C	G - C	G - C	G - C	G - C	G - C	G - C	G - U
	27	177 - 333		U - G	U - G	C - G	C - G	U - G	C - G	C - G	U - G	C - G	C - G	C - G	C - G	C - G	C - G	C - G
	28	178 - 332		G - C	G - C	G - C	G - C	G - C	G - C	G - C	G - U	G - C	G - C	G - C	G - C	G - C	G - C	G - C
	29	179 - 329		G - U	G - C	G - U	G - U	A - U	G - U	G - U	G - U	G - U	G - U	G - U	G - U	G - U	G - C	C - G
	30	180 - 327		G - C	A x C	G - C	G - C	G - U	G - U	G - U	G - C	A - U	G - U	G - C	G - C	G - C	G - C	U - G
	31	181 - 326		C - G	C - G	C - G	C - G	C - G	C - G	C x -	C - G	C - G	C - G	C - G	C - G	C x C	C - G	C - G
	32	182 - 325		G - C	G - C	G - C	G - C	G - U	G - C	G - U	G - C	G - C	G - U	G - U	G - U	G - U	G - C	G - C
	33	183 - 322		C - G	C - G	C - G	C - G	C - G	C - G	C - G	C - G	C - G	C x C	U - G	U - G	U - G	U - G	C - G
	34	188 - 314		U - A	U - G	U - G	U - G	U - G	U - G	U - G	U - G	U - G	C - G	U - G	U - G	U - G	- x G	C x A
	35	191 - 313		U - A	U - A	U - A	U - G	U - A	- x A	U - A	U - A	U - A	A - U	A - U	A - U	C x C	A x G	A x A
	36	193 - 312		C - G	C - G	C - G	C x U	U - A	A x G	C - G	C - G	U - A	C - G	*	*	*	*	*
	37	196 - 310		C - G	C - G	C - G	C - G	C - G	C - G	C - G	C - G	C - G	C - G	G - C	G - C	G - C	- x C	C - G
	38	198 - 308		C - G	C - G	C - G	C - G	C - G	- x G	C - G	C - G	- x G	C - G	*	*	*	*	*
	39	200 - 306		G - C	G - C	G - C	G - C	G - C	- x C	G - C	G - C	G - C	G - C	*	*	*	*	*
IIIa	40	207 - 249		G - C	G - C	G - C	G - C	G - C	*	G - U	G - C	G - C	G - U	C - G	C - G	C - G	C - G	U - A
	41	208 - 248		G - C	G - C	G - C	G - C	G - C	*	G - U	G - C	G - C	G - U	C - G	C - G	A - U	C - G	C - G
	42	209 - 245	CBC in B2	n.c.	n.c	n.c	n.c	n.c	n.c	n.c	n.c	n.c	n.c	C - G	C - G	G - U	G - C	U x U
	43	211 - 243	CBC in B2	n.c	n.c	n.c	n.c	n.c	n.c	n.c	n.c	n.c	n.c	U - A	U - A	G - U	C - G	U - G
	44	213 - 239	CBC in B2	n.c	n.c	n.c	n.c	n.c	n.c	n.c	n.c	n.c	n.c	U - A	U - A	C - G	G x G	U - G
	45	218 - 235	CBC in B2	n.c	n.c	n.c	n.c	n.c	n.c	n.c	n.c	n.c	n.c	U - G	U - G	G - C	C - G	G - C
	46	221 - 232		n.c	n.c	n.c	n.c	n.c	n.c	n.c	n.c	n.c	n.c	C - G	C - G	U - G	C - G	G x G
	47	223 - 231	CBC in B2	n.c	n.c	n.c	n.c	n.c	n.c	n.c	n.c	n.c	n.c	G - C	G - C	U - G	C x C	A x G
IIIb	48	253 - 304		U - G	U - A	U - G	U - G	U - G	U - G	C - G	- x A	U - G	U - G	C - G	C - G	A x G	C - G	U x U
	49	255 - 303	CBC A and B	G - C	G - C	G - C	G - C	G - C	G - C	G - C	G - C	G - C	G - C	C - G	C - G	C - G	C - G	C - G
	50	256 - 302		U - A	U - G	C - G	U - G	C - G	U - A	U - A	U - A	U - A	C - G	G - C	G - C	C x C	G - C	C - G
	51	262 - 301		G - U	G - U	G - C	A - U	G - U	G - U	A - U	G - U	G - U	A - U	G - C	G - C	G - C	G - C	G x G
	52	298 - 365		C - G	C - G	C - G	C - G	C - G	C - G	C - G	C - G	C - G	C - G	C - G	C - G	C - G	C - G	A x G
	53	272 - 289		G - C	G - C	G - U	G - U	G - U	G - U	G - C	G - C	G - U	G - C	G - C	G - C	G - C	G - C	G - C
	54	273 - 287	CBC A and B	C - G	C - G	C - G	C - G	C - G	C - G	C - G	C - G	C - G	C - G	U - A	U - A	U - A	U - A	U - A
	55	274 - 286		A - U	A - U	A - U	A - U	A - U	A - U	A - U	A - U	A - U	A - U	U - A	U - A	U - A	G x G	G x G
	56	276 - 284		G - C	G - C	G - C	G - C	G - C	G - C	G - C	G - C	G - U	G - C	C - G	C - G	C - G	G - C	G - C
	57	277 - 282		G - U	- x U	U - A	A - U	G - U	U - A	C - G	A - U	C - G	G - U	U - G	U - G	U - G	A - U	A- U
IV	58	340 - 366		U - G	U - G	C - G	U - G	U - G	C - G	U - A	U - G	C - G	U - G	G - C	G -C	G x G	G - C	C - G

* pair missing

n.c (nom comparable region)

Supplementary figure legends

Supplementary Figure 1: Average cell size (A) and DNA content (B) estimated for 41 strains of Chloropicophyceae. A) Average cell size with standard deviation for each strain from 100 randomly chosen cells. B) Genome size estimated by flow cytometry. *Micromonas commoda* (RCC299) was used as an internal standard (genome size = 21 Mbp).

Supplementary Figure 2: Picocystophyceae, TEM-graph of thin section and SEM-graph. A-B. *Picocystis salinarum* (RCC3402).

Supplementary Figure 3: Maximum-likelihood tree inferred from ITS2 sequences belonging to Chloropicophyceae strains. Solid dots correspond to significant support (>0.7) for ML analysis and full support (1.0) by Bayesian analysis. When ML support is below 1.0 the percentage is indicated next to the symbol. Grey dots correspond to non-significant ML support (<0.7) and full support from Bayesian analysis. Empty dot corresponds to ML support without support from Bayesian analysis.

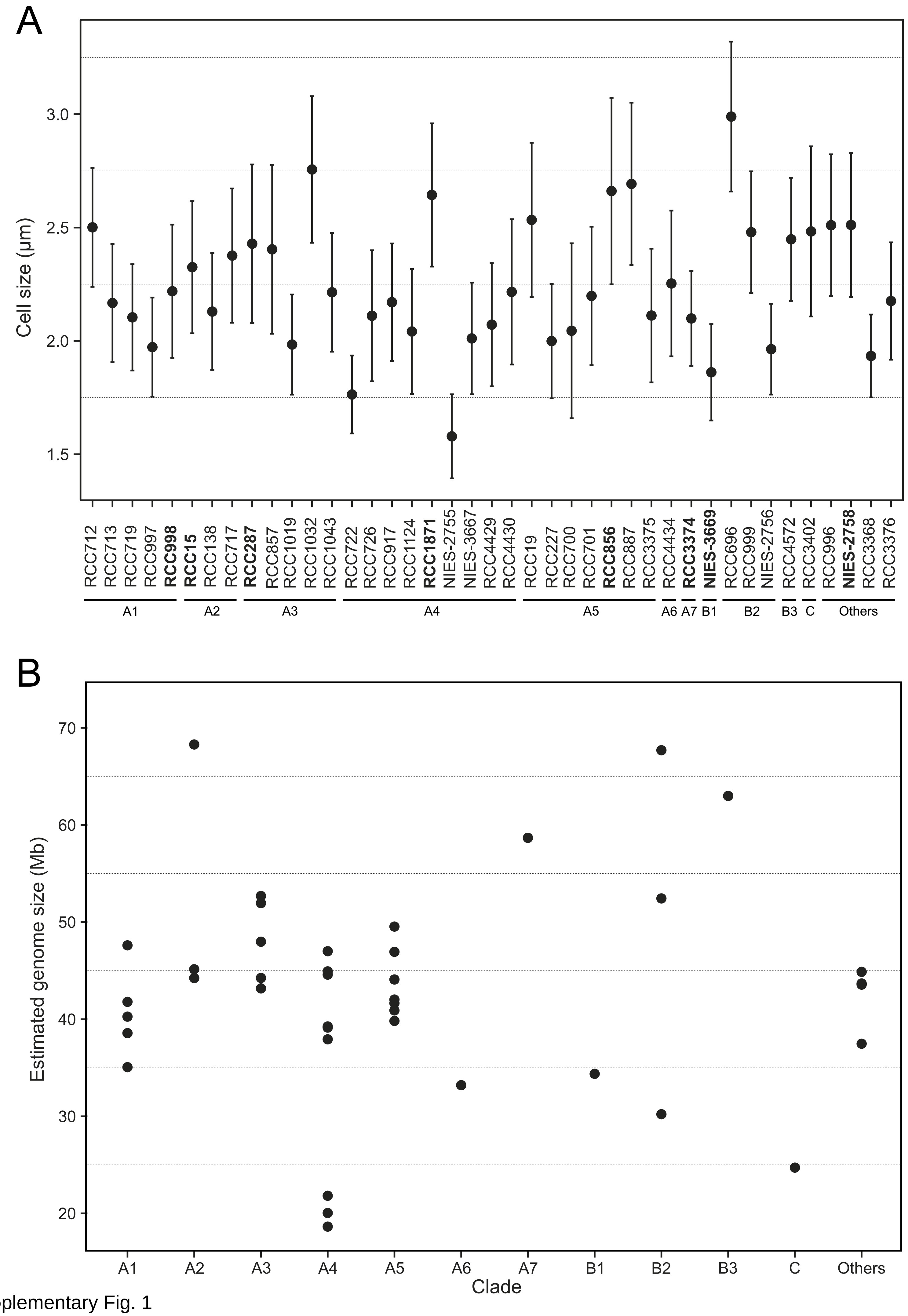

Supplementary Fig. 1

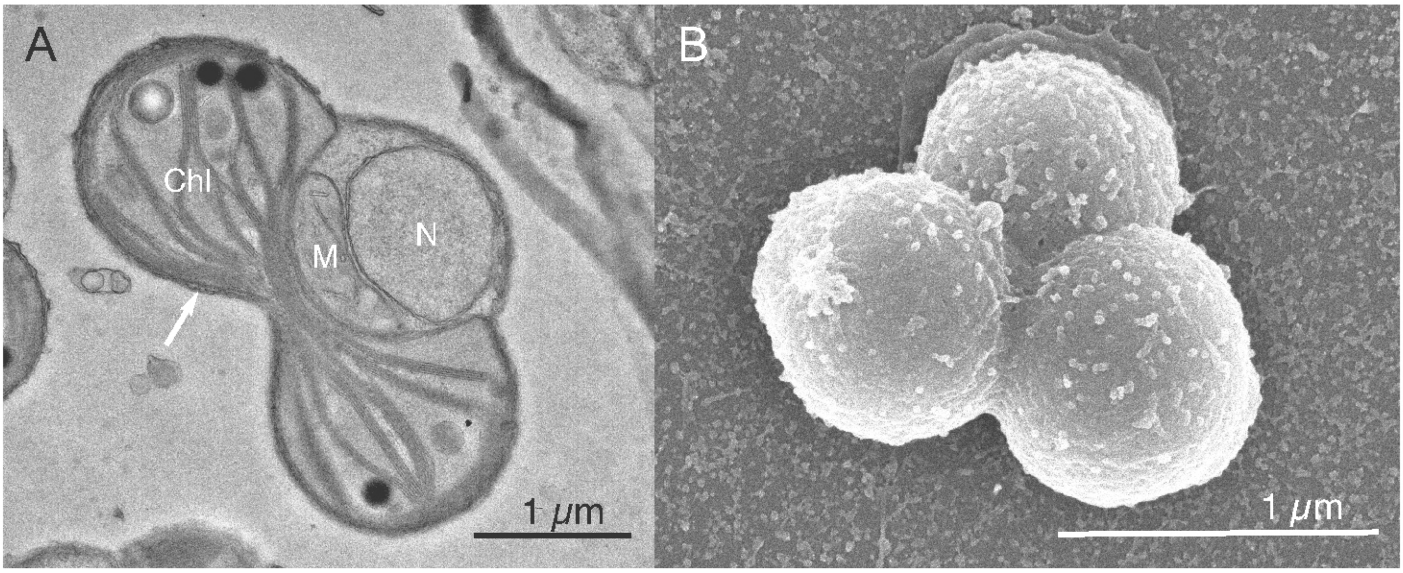

Supplementary Fig. 2

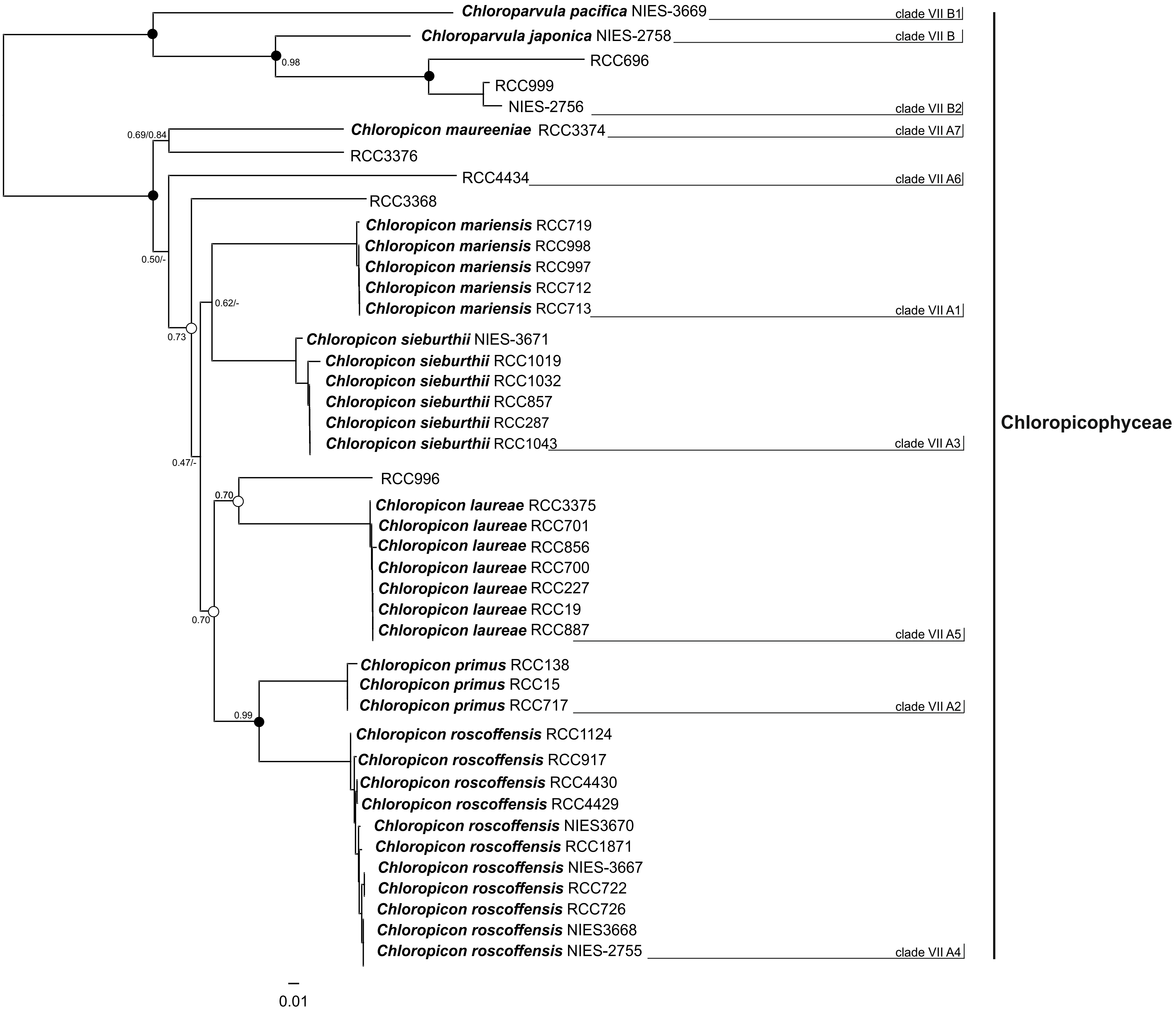

Supplementary Fig. 3

List of Supplementary Material

All supplementary material including data, figures and tables are available from https://doi.org/10.6084/m9.figshare.5027375

Supplementary Material 1: ITS secondary structure data file in Vienna format.

Supplementary Material 2: Concatenated alignment for 18S and 16S gene.

Supplementary Material 3: Concatenated alignment for transcriptomes.
